# Dynamic changes of soil microorganisms in rotation farmland at the western foot of the Greater Khingan range

**DOI:** 10.3389/fbioe.2023.1191240

**Published:** 2023-06-23

**Authors:** Shuli Wei, Jing Fang, Tianjiao Zhang, Jianguo Wang, Yuchen Cheng, Jie Ma, Rui Xie, Zhixiong Liu, Erhu Su, Yongfeng Ren, Xiaoqing Zhao, Xiangqian Zhang, Zhanyuan Lu

**Affiliations:** ^1^ School of Life Science, Inner Mongolia University, Hohhot, China; ^2^ Inner Mongolia Academy of Agricultural and Animal Husbandry Sciences, Hohhot, China; ^3^ Key Laboratory of Black Soil Protection and Utilization (Hohhot), Ministry of Agriculture and Rural Affairs, Hohhot, China; ^4^ Inner Mongolia Key Laboratory of Degradation Farmland Ecological Remediation and Pollution Control, Hohhot, China

**Keywords:** rotation patterns, drought stress, bacteria, fungi, soil factors

## Abstract

Crop rotation and other tillage systems can affect soil microbial communities and functions. Few studies have reported the response of soil spatial microbial communities to rotation under drought stress. Therefore, the purpose of our study was to explore the dynamic changes of the soil space microbial community under different drought stress-rotation patterns. In this study, two water treatments were set up, control W1 (mass water content 25%–28%), and drought W2 (mass water content 9%–12%). Four crop rotation patterns were set in each water content, spring wheat continuous (R1), spring wheat-potato (R2), spring wheat-potato-rape (R3) and spring wheat-rape (R4), for a total of eight treatments (W1R1, W1R2, W1R3, W1R4, W2R1, W2R2, W2R3, W2R4). Endosphere, rhizosphere and bulk soil of spring wheat in each treatment were collected, and root space microbial community data were generated. The soil microbial community changed under different treatments and their relationship with soil factors were analyzed using a co-occurrence network, mantel test, and other methods. The results revealed that the alpha diversity of microorganisms in the rhizosphere and bulk soil did not differ significantly, but it was significantly greater than in the endosphere. The bacteria community structure was more stable, fungi alpha-diversity significant changes (*p* < 0.05), that were more sensitive to the response of various treatments than bacteria. The co-occurrence network between fungal species was stable under rotation patterns (R2, R3, R4), while the community stability was poor under continuous cropping pattern (R1), and interactions were strengthened. Soil organic matter (SOM), microbial biomass carbon (MBC), and pH value were the most important factors dominating the bacteria community structural changed in the endosphere, rhizosphere, and bulk soil. The dominant factor that affected the fungal community structural changed in the endosphere, rhizosphere, and bulk soil was SOM. Therefore, we conclude that soil microbial community changes under the drought stress-rotation patterns are mainly influenced by soil SOM and microbial biomass content.

## 1 Introduction

Black land is a precious natural resource and the most valuable soil for humans. It is critical in improving food production and maintaining food security (Wang et al., 2022). The Greater Khingan Mountains’ western foot is a typical representation of black land in the Inner Mongolia Autonomous Region. Recently, problems such as thinning of the black soil layer and reduction of organic matter have occurred due to reuse, light tillage, and soil erosion. For example, fertility and permeability have decreased (Fang and Fan, 2020; Ma et al., 2023). Cultivated land quality improvement plays an important role in protecting and utilizing black land. Crop rotation combined with reasonable tillage measures can effectively protect and improve the soil environment, affect the diversity of soil microorganisms, and thus improve soil productivity and crop yield (Gong et al., 2021; Volsi et al., 2022; Zhou et al., 2023).

Recently, the precipitation has decreased in the west foot of the Greater Khingan Mountains, and drought has occurred annually, affecting crop growth and development, resulting in a 20%–30% reduction in annual yield. The agricultural production loss is huge, and it has become the primary obstacle limiting agricultural production development in the region (Jiao et al., 2020). Soil moisture is the key factor affecting crop growth. Nutrient availability, the mineralization reaction process, and the effectiveness of nitrogen decrease in the soil as soils become drier (Schuur and Matson, 2001; Larsen et al., 2011; Hartman and Tringe, 2019). Drought stress significantly reduces soil phosphorus availability and plant absorption of available phosphorus (Suriyagoda et al., 2011). The soil’s physical contents and chemical and microbiological properties, under continuous cropping and rotation, are also the primary factors affecting crop growth and development. The soil is prone to form continuous cropping obstacles, excessive consumption of soil nutrients, deterioration of soil physical and chemical properties, an increase of pests and diseases, and toxic substance accumulation under continuous cropping pattern (Tan et al., 2021), resulting in crop growth and development damage, reduced photosynthetic capacity and biomass accumulation, and susceptibility to diseases. It eventually reduces soil productivity and yield (Wang et al., 2020). Rotation patterns can increase the soil water storage capacity and moisture conservation, improve soil nutrient movement and the coordination of water, fertilizer, air, and heat, increase the soil nutrient content, improve soil enzyme activity, soil microbial biomass, carbon, nitrogen, and phosphorus content, fertilize the soil, change the soil microenvironment, and achieve high crop yield (Haruna and Nkongolo, 2019; Shi, 2021).

Soil microorganisms strongly affect the interaction between plants and soil (Miethling et al., 2000), converting organic matter into mineral elements. The interaction between crops, soil microenvironment, and soil microorganisms jointly maintains the balance and multiple functions of the terrestrial ecosystem (Leff et al., 2015; Delgado-Baquerizo et al., 2017). Drought significantly affects bacterial and fungal communities and is largely attributable to changes in plant cover and subsequent feedbacks on soil physicochemical properties (especially pH) (Seaton et al., 2022). Drought promotes unstable properties in symbiotic networks of soil bacteria (but not fungi) (de Vries et al., 2018), affects the complexity and stability of bacterial communities. Changes in precipitation will also significantly reshape bacterial interactions in semi-arid grasslands (Wang et al., 2022). The different rotation and fallow patterns impact the microbial community structure of spring wheat spatial location under natural conditions. Compared with continuous cropping pattern, the Alpha diversity index of soil microorganisms in spring wheat fields under rotation fallow pattern is higher, with rich species and relatively stable community structure (Shi, 2021). Mulching treatment and fertilizer treatment also significantly impact the soil microbial community structure at the spatial location of crop roots (Francioli et al., 2016; Sun et al., 2020; Wang et al., 2022; Yca et al., 2020), microbial communities in sorghum phyllosphere and root endosphere are more resistant than soil microbiota to long-term fertilization, and soil microbiota are important predictors of sorghum yield and protein content. Meanwhile, wheat variety, location, and growth stage significantly impact the interaction of microbial community assembly in the root space (Zheng et al., 2021). Microbial communities were more diverse in the bulk soil and rhizosphere than in root endosphere. Wheat-root associated microbial community assembly was shaped predominantly by different factors while within each factor, location had stronger effects on the variation in prokaryotic community than growth stage or variety. Similarly, crops affect the soil microbial community and nutrients in the farmland ecosystem (Gautier et al., 2020). Simultaneously, microbial substrate decomposition, energy transfer, and nutrient cycling adversely affect crop development (Stefan et al., 2021; Xie et al., 2022). However, there are fewer studies on the trends of microbial changes in spring wheat roots under different drought stress-rotation pattern treatments and their main drivers. Therefore, we explored the relationship between the microbial community in the root space of spring wheat and soil properties under the drought stress-rotation patterns to clarify the potential causes of soil nutrient-driven microbial changes under rotation patterns.

Our study examines the comprehensive effects of water, soil factors, and rotation patterns in microbial communities, focusing on the crucial role of different soil factors in shaping microbial communities. In the experiment, we use Longmai36 as the test material, set two moistures and four rotation patterns; thus, eight treatments in total. We collected roots, rhizosphere, and bulk soils from spring wheat, generated microbial community structure data for root space, and conducted quantitative characterization of microbial community composition and structure. It is helpful to understand the continuous characteristics of bacterial and fungal community changes in soil space under different drought stress-rotation patterns. Therefore, we propose the following assumptions: 1) drought stress-rotation patterns can significantly affect wheat root space community composition and microbial symbiosis patterns; 2) soil nutrient is the primary environmental factor affecting the microbial community, while drought stress and rotation patterns are indirect factors.

## 2 Materials and methods

### 2.1 Study area

The test site was at the Tenihe River Scientific Observation and Test Station, Inner Mongolia Academy of Agriculture and Animal Husbandry Sciences (Tenihe Farm E 120° 48′, N 49° 55′, 650 m above sea level). The climate zone was semi-arid continental grassland in the middle temperate zone. The annual average temperature was 2.2°C, the frost-free period was 90–105 days, the average yearly precipitation was 373–474 mm, and the precipitation from May to August 2022 was 184.5 mm. The cultivated land was chernozem at the experimental site, with a field water capacity of 38%, and characteristic of the black soil near the west foot of the Greater Khingan Mountains in Inner Mongolia’s typical farming-pastoral ecotone.

### 2.2 Experimental design

**FIGURE 1:**
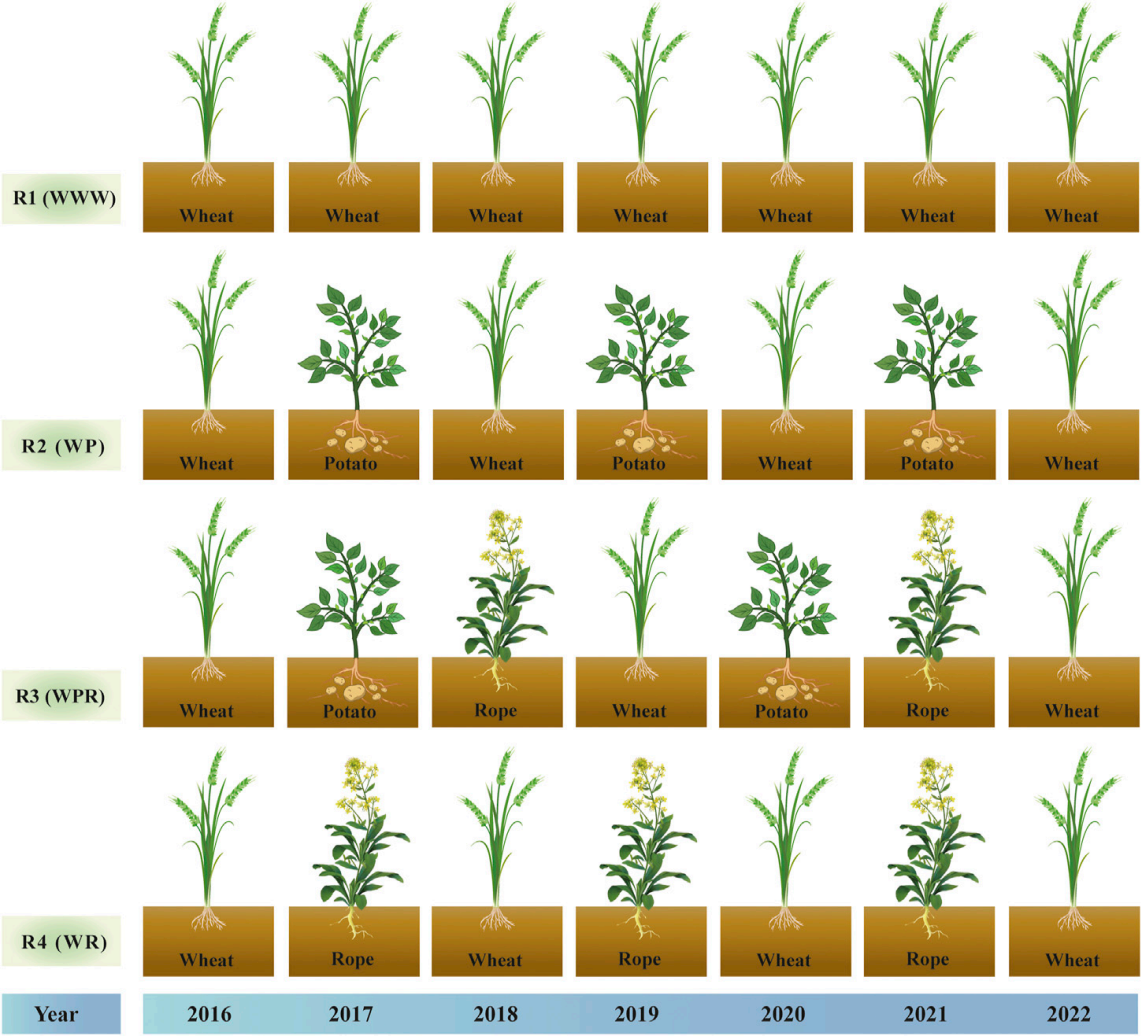
Crop rotation sequence diagram from 2016 to 2022: R1 (WWW) represents spring wheat continuous cropping, R2 (WP) represents spring wheat-potato, R3 (WPR) represents spring wheat-potato-Rope and R4 (WR) represents spring wheat-Rope, respectively.

**FIGURE 2:**
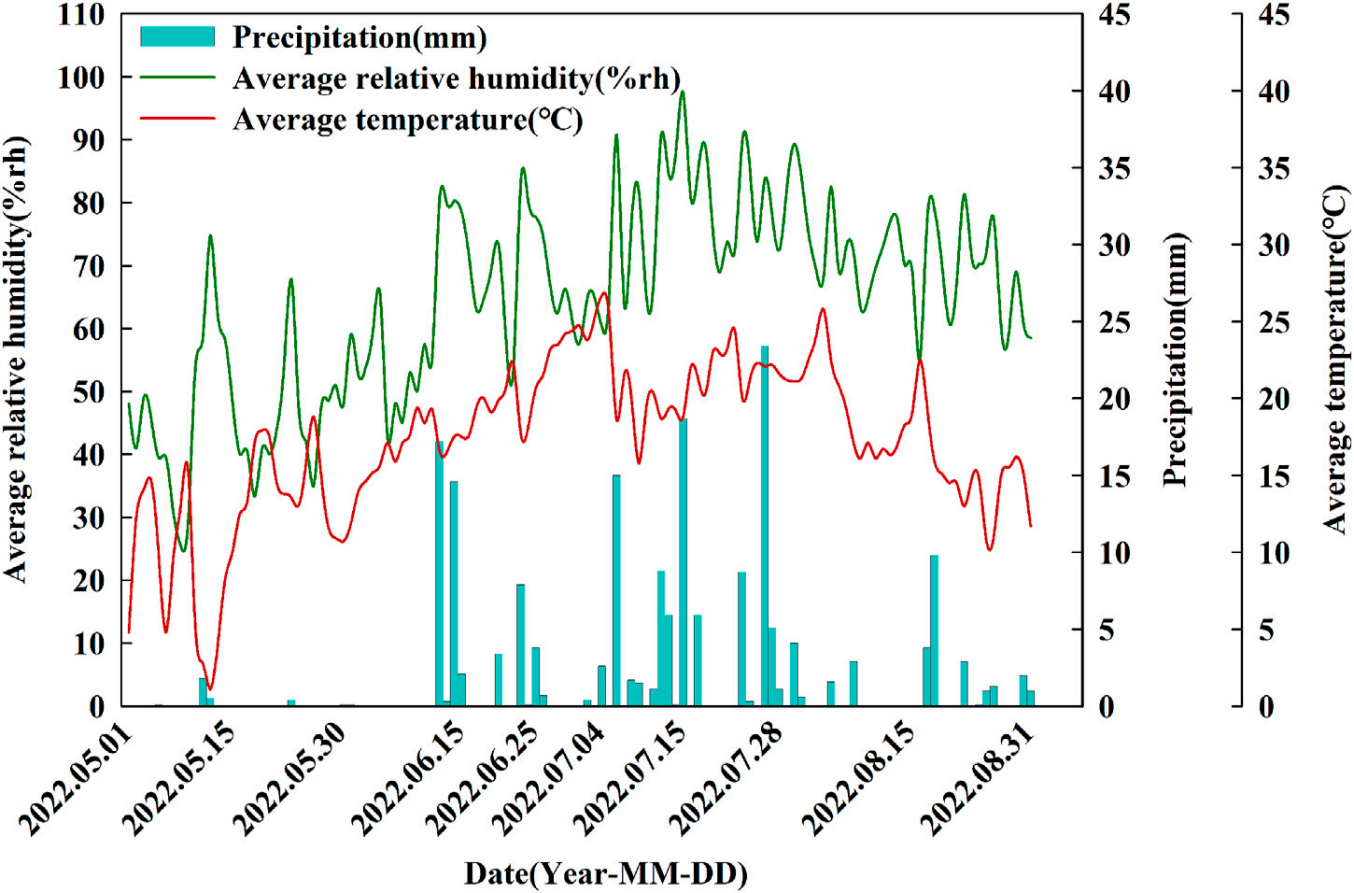
Dynamics of the daily average relative humidity, precipitation and average temperature during the experimental period from May to August.

This two-factor experiment involved four rotation patterns and two moisture treatments. The primary factor was rotation over 6 years with four designs, spring wheat continuous (R1), spring wheat-potato (R2), spring wheat-potato-rape (R3) and spring wheat-rape (R4). The secondary factor was mass water content at the flowering stage of wheat, including control treatment W1 (25%–28%) and drought stress treatment W2 (9%–12%) (Hsiao, 1973). Four rotation patterns were positioned starting in 2016, and after a 6-year rotation, spring wheat was planted and sampled uniformly in year 7 (2022). We studied the first 6 years of the crop rotation as a whole. Figure 1 depicts the crop planting sequence for the crop rotations. The water treatment started at the late stage of wheat jointing stage (July 4). End of late stage of wheat flowering on 27 July 2022. Control and drought treatment uses plastic greenhouses to control moisture. The two sides of the plastic greenhouse are 1.5 m above the ground to maintain ventilation of the wheat. The drought treatment was kept without rehydration until the soil mass moisture content of the continuous crop pattern was reduced to 9%–12% on 27 July 2022, processing completed. The soil moisture content of control treatment was kept at 25%–28%, and replenished water manually during this period. The irrigation amount was fixed at 900 m^3^/ha each time. R1 and R4 were irrigated manually on July 8 and July 24, and R1 and R3 were irrigated manually on July 21 and July 24. Both control and drought resistance treatments were sampled simultaneously on 28 July 2022 (Figure 2).

Longmai36 is a spring wheat variety characterized by its high yield, high quality, multi-resistance, wide adaptability, drought tolerance, and cold tolerance. It is suitable for planting in eastern Heilongjiang Province, the western foothills of the Greater Khingan Mountains, and the northwest foothills of Daxing’an Ridge, with a planting density of three million plants per hectare and 15 cm row spacing. The study included two moisture and four rotation patterns treatments; thus, eight treatments: W1R1, W1R2, W1R3, W1R4, W2R1, W2R2, W2R3, and W2R4; each with three replicates. Each subplot was 9 m^2^ (6 × 1.5 m) in area, with a buffer zone of 1 m wide between each subplot, uniform base fertilizer per treatment, urea 60 kg/ha, ammonium dihydro phosphate 165 kg/ha, and potassium sulfate 30 kg/ha. Before sowing, the rotary tillage was applied uniformly to the 0–15 cm soil layer, and other management methods were the same as field production. Canola and potatoes were included in the rotation patterns. Wheat and rape were cultivated without tilling, while potatoes were tilled. Approximately 50%–60% of wheat straw was returned to the field, and all rape and potato straws were returned. Table 1 illustrates specific fertilization.

**TABLE 1:** Management information of crops in the experiment.

Crop	Varity name	Row spacing (cm)	Seeding rate (kg/ha)	Seed spacing (cm)	Fertilizer rate (kg/ha)				Tillage method	Straw treatment
					Nitrogen (CH_4_N_2_O)	Phosphorus ((NH₄) ₂HPO₄)	Potassium (K₂SO₄)	Boron (Na_2_B4O_7_		
								·10H_2_O)		
Wheat	Longmai36	15	20	—	60	60	30		No-tillage e	50%-60%-returning
Rope	Qingza5	30	6	—	60	165	30	3	No-tillage	Total-returning
Potato	Xingjia2	65	—	30	225	225	90		Ploughing-tillag	Total-returning

### 2.3 Soil sampling

The sampling time was 10:00 a.m. on 28 July 2022. A total of 144 samples (8 treatments × 3 positions × 6 replicates (3 for sequencing, 3 for retention of samples) = 144 samples) were collected from three different root spaces under eight different combinations of spring wheat in the dry shed at the late flowering stage (microorganisms in the intercellular fluid inside the root), the rhizosphere (microorganisms contained in the soil within 1 mm from the root surface), and the bulk soil (microorganisms contained in the soil 1 mm larger than the root surface). In order to prevent sample loss and other situations during 16s and ITS sequencing, 72 of the 144 samples collected were stored in the laboratory’s ultra-low temperature refrigerator, and the sequencing data of the 72 samples were analyzed in this paper. Sampling tools were disinfected before sampling. Bulk soil samples were collected, wheat that grew evenly between two rows of wheat was selected, and soil mulch was removed. The PVC pipe was driven 0–20 cm into the soil; the five-point sampling method was used for the same treatment. The mixed soil samples were collected, coarse roots, stones, and soil animals were removed, and the soil was screened (1 mm) as bulk soil samples and packed into six sterile pipes. The remaining bulk soil was sieved through a 1-mm sieve, air-dried, and stored for chemical property and enzyme activities measurement. Rhizosphere sample was collected using a shovel to dig out the spring wheat root of 0–20 cm soil layer and shake, or a large piece of soil was removed using a sterile brush. Approximately 1 mm of soil was attached to the root; the sample was placed on the ice and transported back to the laboratory. A sterile brush was used to collect the residual soil within 1 mm of the root surface and screen the soil sample (1 mm) as the rhizosphere soil sample. The root sample was collected and placed on ice before being rinsed with sterile water for 0.5 min, 75% ethanol for 1 min, 2% NaClO for 3 min, 75% sterile ethanol for 1 min, and sterile water for 0.5 min. Then, the surface sterile plant tissue was placed in a centrifuge tube. After dividing the samples into centrifuges, they were frozen with liquid nitrogen and transferred to −80 °C refrigerators for testing soil microorganisms (Mendes et al., 2014; Bai et al., 2022).

### 2.4 Soil physiochemical analysis

The soil pH was measured using potentiometry. Soil organic matter (SOM) content was determined using oxidation dilution of potassium dichromate (Nelson, 1982). Effective available nitrogen (AN) content was determined using the Kjeldahl method (Kjeltec 8400, FOSS Corporation, Denmark), the total phosphorus (TP) content was determined using the sulfuric acid-perchloric acid digestion method (Uv-vis TU-1810, Persee, Beijing), the total potassium (TK) was determined using China hydroxide (NaOH) melting flame photometric (Flame photometer Ap1302, AOPU, Shanghai) (Bao, 2000). The soil microbial biomass carbon (MBC) and soil microbial biomass nitrogen (MBN) were determined using chloroform fumigation, soil microbial biomass phosphorus (MBP) content was determined using the total phosphorus determination method (Uv-vis TU-1810, Persee, Beijing) (Vance, 1987). The soil sucrase activity was determined using 3,5-dinitro salicylic acid colorimetry, the soil urease activity (UE) was determined using indophenol blue colorimetry, the soil urease activity was determined using phenol-sodium hypochlorite colorimetry, the soil alkaline phosphatase (ALP) activity was determined using diphenyl phosphate colorimetry, the soil catalase (CAT) content was determined using potassium permanganate titration (Uv-vis TU-1810, Persee, Beijing) (Guan, 1986). The soil moisture content was determined using the ring knife method and calculated using the formula: soil moisture content (%) = (WWT-WDry)/WDry×100% (Zhang et al., 2005).

### 2.5 DNA extractions, sequencing, and data processing

Wheat root and soil DNA were extracted according to the manufacturer’s instructions using the E.Z.N.A.^®^ soil DNA Kit (Omega Bio-tek, Norcross, GA, U.S.). The DNA extract was examined using 1% agarose gel, and DNA concentration and purity were determined using NanoDrop 2000 UV-vis spectrophotometer (Thermo Scientific, Wilmington, United States). The hypervariable region V3-V4 of the bacteria 16S rRNA gene was amplified with primer pairs 338F (5′-ACT​CCT​ACG​GGA​GGC​AGC​AG-3′) and 806R (5′-GGACTACHVGGGTWTCT AAT-3′) using an ABI GeneAmp^®^ 9700 PCR thermocycler (ABI, CA, United States). The ITS1-ITS2 region of fungal rRNA was amplified by PCR using the universal primers ITS1F (CTT​GGT​CAT​TTA​GGA​AGT​AA) and ITS2 (GCT​GCG​TTC​TTC​ATC​GAT​GC). Purified amplicons were pooled in equimolar, and paired-end sequenced using an Illumina NovaSeq PE2500 platform (Illumina, San Diego, United States) according to the standard protocols by Majorbio Bio-Pharm Technology Co., Ltd. (Shanghai, China). The raw reads were deposited to the NCBI Sequence Read Archive (SRA) database (Accession Number: PRJNA943477). Operational taxonomic units (OTUs) with a 97% similarity cutoff (Stackebrandt and Goebel, 1994; Edgar, 2013) were clustered using UPARSE version 7.1 (Edgar, 2013), and chimeric sequences were identified and removed. The taxonomy of each OUT representative sequence was analyzed using RDP Classifier version 2.2 (Wang et al., 2007) against the database (16S: Silva v138 and ITS: unite8.0/its_fungi) with a confidence threshold of 0.7.

### 2.6 Statistical analysis

Qiime 1.9.1 is used to generate taxonomic abundance tables and beta diversity distance calculations. Mothur 1.30.2 was used to calculate microbial alpha diversity. Linear mixed model (LMM) was used to analyze the effect of drought stress-rotation patterns treatment and spatial structures on soil properties, enzymatic activities, Alpha diversity, microbial phyla. Statistical analysis was conducted using SPSS Statistics 17.0 software (SPSS Inc., Armonk, NY, United States). The following statistics were conducted in version R3.3.1. The differences in the composition of microbial communities were analyzed the principal component analysis (PCoA) based on the Bray-Curtis dissimilarity matrix. The effects of drought stress-rotation pattern treatments on changes in bacterial and fungal composition were analyzed based on PERMANOVA using the R vegan pack. Redundancy analysis (RDA) was used to identify soil factors that significantly affect wheat root’s bacterial and fungal community spatial, using RDA analysis and mapping in vegan package. Correlations between environmental factors and species are analyzed using a heatmap package. A partial Mantel test and variation partitioning analysis (VPA) was used to determine the relative contribution of soil moisture, rotation patterns, and soil factors to microbial community variation, analysis software is VPA analysis in vegan package. Aggregated boosted tree (ABT) analysis (De’ath, 2007; Wang et al., 2020) was performed using the gbmplus package (with 500 trees used for the boosting, 0.02-fold shrinkage rate, and three-way interactions) to determine the relative influence of environmental variables on bacteria and fungi community composition (PCoA axis 1).

To compare the bacterial and fungal co-occurrence networks between different rotation patterns, we combined two moisture treatments from the same rotation pattern. Gephi (version 0.9.7; https://gephi.org/) was used for network visualization (Bastian et al., 2009). The data are based on OTU tables, calculated using the R language package Networkx. The correlation coefficient is Spearman correlation coefficient. The top 100 OTUs in terms of abundance were selected for co-occurrence networks construction. The nodes in the network represented OUTs, while the edges represented the correlation between two OTUs. The combinations with correlation coefficient (*ρ*) were kept greater than 0.8 and *p* < 0.05. The thickness of each edge was proportional to the value of Spearman’s correlation coefficient. The networks’ topological features, such as modularity, average degree, and average clustering coefficient, were used to describe the network structure (Barberán et al., 2012). Modularity index measures the degree of modularity of the network graph structure, generally >0.4 indicates that the network graph has achieved a certain degree of modularity (Bi et al., 2021); Diameter is the distance between two nodes in the network as the number of edges on the shortest path connecting the two nodes; The maximum value of the distance between any two nodes in the network is called the diameter of the network; Average degree denotes the average number of edges connected to each node; Graph density is the ratio of the number of edges actually present in the network to the upper limit of the number of edges that can be accommodated; The average path length is defined as the average number of steps along the shortest paths for all possible pairs of network nodes; The degree indicates the number of nodes directly connected to the node in the network, and the higher the connectivity means the higher the importance of the node in the whole network, a node with very high connectivity is also called a critical node (Newman, 2003).

## 3 Results

### 3.1 Changes in microbial alpha diversity

We identified 3,083,027 bacterial and 3,069,099 fungal high-quality sequences in the root endosphere; 3,560,336 bacterial and 3,141,508 high-quality fungal sequences in the rhizosphere soil; and 3,875,463 bacterial and 3,166,993 high-quality fungal sequences in the bulk soil (Supplementary Table S1). The bacterial and fungal sequences were clustered into 7,370 and 2090 OTUs in the endosphere, 12,891 and 4,602 OTUs in the rhizosphere soil, and 13,366 and 4,597 OTUs in the rhizosphere soil, respectively (Supplementary Table S1).

Alpha diversity of bacterial and fungal communities in rhizosphere soil and bulk soil exhibited a significantly higher rate of change than endosphere bacterial and fungal communities (Supplementary Table S2). Different drought stress-rotation pattern treatments induced significant (*p* < 0.01) changes in the Shannon index, Chao1 estimator of the bacterial community in bulk soil, rhizosphere, and fungal community in the rhizosphere (Supplementary Table S2). The W1R1 treatment resulted in the highest Shannon index and number of observed OTUs in samples of endosphere fungi. The Chao1 estimator among bacteria samples from the endosphere and the Shannon index, Chao1 estimator among fungi samples were highest from the bulk soil under the W2R3 treatment. The W2R4 treatment produced the highest Shannon index and Chao1 estimator among bacterial and fungal rhizosphere samples (Supplementary Table S2).

### 3.2 Microbial community composition

**FIGURE 3:**
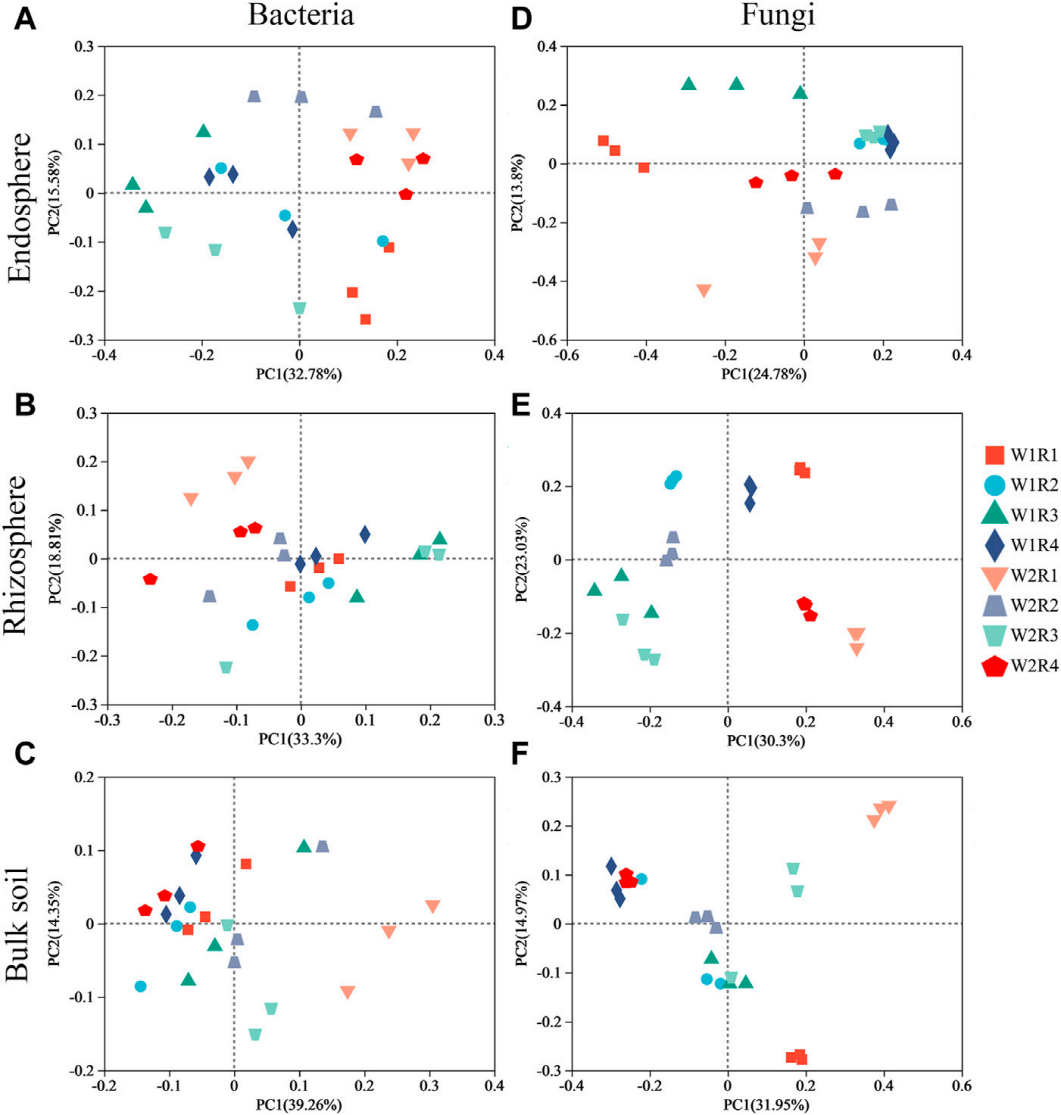
Ordination of root space microbial community (OTU abundance) in wheat soil with different drought stress-rotation patterns using PCA with the Bray−Curtis similarity index. **(A–C)**: endosphere, rhizosphere, bulk soil bacteria; **(D–F)**: endosphere, rhizosphere, bulk soil fungi.

Space structure influences bacterial and fungal community structures (Supplementary Figures S1A, B). The fungal community composition in the rhizosphere, rhizosphere, and bulk soil was more discrete than the bacterial community composition (Figure 3), suggesting that drought stress-rotation treatment has a greater impact on the composition of the fungal communities than bacteria. According to PERMANOVA analysis, treating drought stress, rotation patterns, and drought stress-rotation patterns resulted in significant changes in the fungal community but not in the bacterial community. Additionally, drought stress-rotation pattern treatment strongly affected the rhizosphere (*R* ^2^ = 0.88) and bulk soil (*R* ^2^ = 0.76) fungal communities’ composition. Drought stress rotation pattern treatment had a greater impact on endosphere bacteria communities (*R* ^2^ = 0.67) than on fungal communities (*R* ^2^ = 0.63) (Table 2).

**TABLE 2:** Effects of drought stress-rotation patterns treatment on the changes of bacterial and fungal composition based on PERMANOVA.

Type	Kingdoms	PERMANOVA	
		*R* ^2^	*p*
Endosphere	Bacteria	0.67	0.001
	Fungi	0.63	0.001
Rhizosphere	Bacteria	0.57	0.001
	Fungi	0.88	0.001
Bulk soil	Bacteria	0.61	0.001
	Fungi	0.76	0.001
Spatial structure	Bacteria	0.67	0.001
	Fungi	0.26	0.001
Soilmoisture	Bacteria	0.022	0.181
	Fungi	0.056	0.002
Rotation	Bacteria	0.041	0.447
	Fungi	0.14	0.001
Droughtstress−Rotationpattern	Bacteria	0.09	0.567
	Fungi	0.26	0.001

**FIGURE 4:**
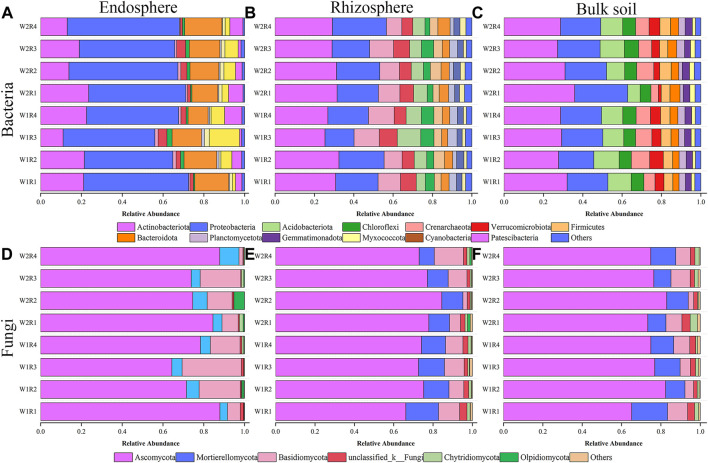
Relative abundance of the taxonomic composition of endosphere, rhizosphere, bulk soil, and bacteria **(A–C)**, and fungal **(D–F)** community at phylum level under different drought stress-rotation patterns treatment, respectively.

Drought stress-rotation pattern treatment dramatically affected the microbial community composition of the whole root spatial structure. The three most abundant bacteria detected in the endosphere were Proteobacteria (43.43%–55.04%), Actinobacteria (11.02%–23.54%), and Bacteroidota (9.87%–18.21%). The treatments with the highest relative abundance corresponding to the above three dominant phyla were W2R1, W2R4, and W1R3 (Figure 4A; Supplementary Table S3). Actinobacteriota (25.35%–32.48%, 27.21%–32.05%), Proteobacteria (14.81%–27.49%, 17.92%–26.99%), and Acidobacteriota (7.82%–13.05%, 6.30%–12.90%) were the abundant bacteria in the rhizosphere and bulk soil. W1R2, W2R4, and W1R3 were treated for the three most abundant relative phyla. W1R2, W2R4, and W1R3 treatments had the highest abundance of the three dominant phyla corresponding to the rhizosphere. The treatments with the highest abundance corresponding to the bulk soil were W2R1, W2R1, and W1R2 (Figures 4B, C; Supplementary Table S3).

The three most abundant fungi were Ascomycota (64.33%–87.82%, 66.18%–84.42%, 65.12%–82.97%), Mortierellomycota (3.70%–9.45%, 7.69%–16.62%, 10.98%–18.29%), and Basidiomycota (1.95%–29.01%, 2.17%–14.83%, 2.73%–10.18%) in the endosphere, rhizosphere, and bulk soil, respectively. The treatments with the highest relative abundance of endosphere, rhizosphere, and bulk soil corresponding to the above three dominant phyla were W2R4, W2R4, W2R3, W2R1, W2R1, W2R4, W2R2, W2R1, and W2R1, respectively (Figures 4D–F; Supplementary Table S3).

Drought stress-rotation patterns dramatically affect the abundance of *f__Mitochondria*, *Lechevalieria*, *TM7a*, *Chitinophagaceae*, *Pseudoxanthomonas*, *Flavobacterium*, *f__Micromonosporaceae* in the endophytic environment; *f__Nitrososphaeraceae*, *Bacillus,* and *Sphingomonas* in rhizosphere soil; and the *f__67−14*, *Sphingomonas*, *Candidatus_Udaeobacter* in bulk soil (Supplementary Figure S2). The most abundant bacterial genus was *f__Mitochondria* (3.23%–19.07%) in root endophytic environment, and *f__Nitrososphaeraceae* (3.53%–9.25%, 2.90%–7.13% in the rhizosphere and bulk soil. The most abundant fungal genera were *Parastagonospora* (0.74%–42.55%) in the endosphere of the root, *Mortierella* (7.11%–16.32%) in rhizosphere soil, and *Mortierella* (8.57%–18.25%) and *Trichocladium* (0.39%–23.94%) in bulk soil. Furthermore, drought stress-rotation patterns caused a significant change in the abundance of *Parastagonospora*, *f__Lasiosphaeriaceae*, *c__Sordariomycetes*, *Schizothecium*, *Penicillium*, *Neosetophoma*, *Vishniacozyma* in endosphere; *Mortierella*, *Gibberella*, and *Fusicolla* in the rhizosphere soil; and *Mortierella* and *Fusarium* in the bulk soil (Supplementary Table S4).

### 3.3 Microbial co-occurrence networks

**FIGURE 5:**
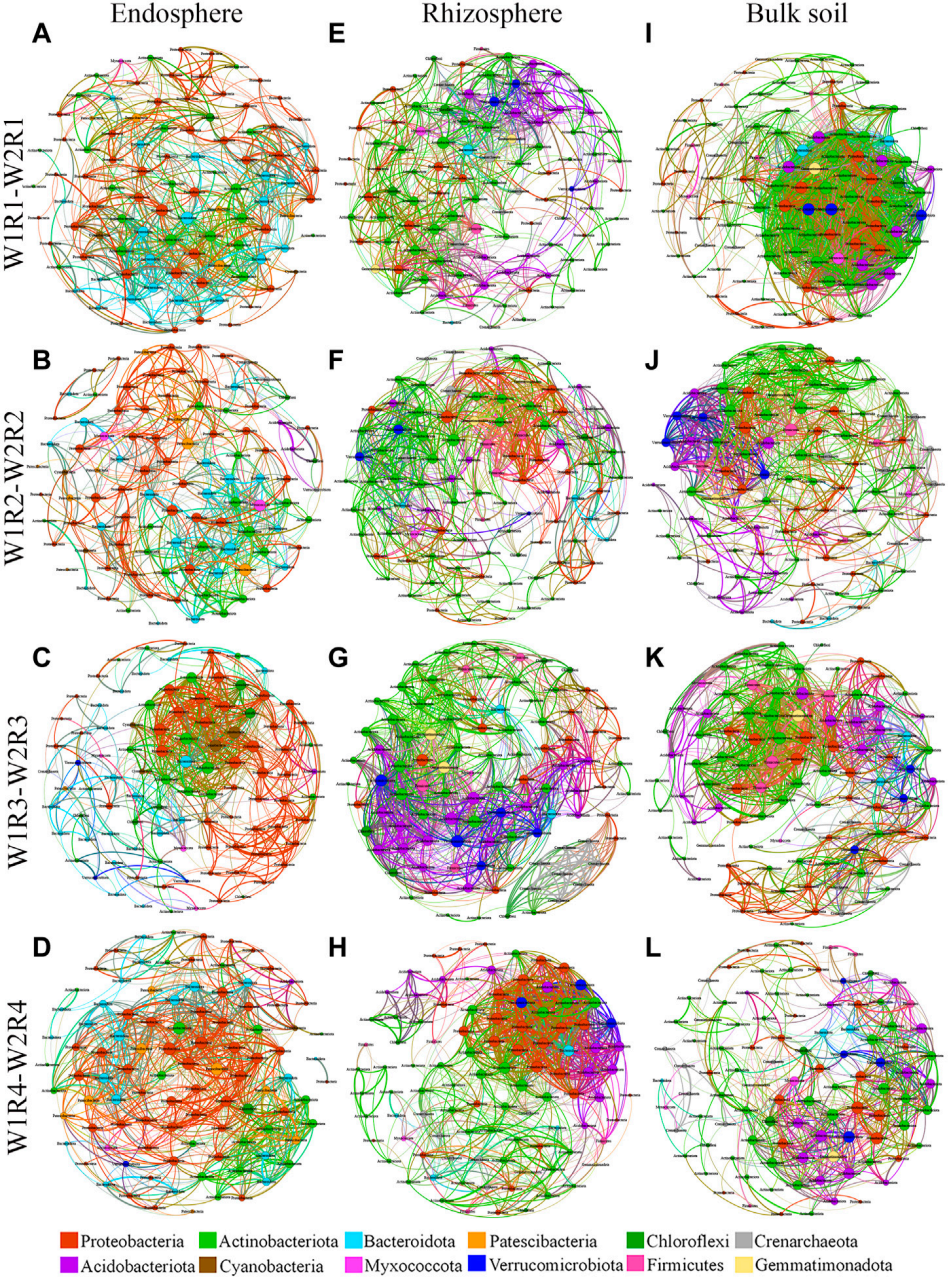
Soil bacteria co−occurrence networks of different drought stress−rotation patterns, including endosphere, rhizosphere, and bulk soil. **(A,E,I)** W1R1-W2R1; **(B,F,J)** W1R2-W2R2; **(C,G,K)** W1R3-W2R3; **(D,H,L)**: W1R4-W2R4). Connections indicate significant correlation (Screening conditions: Spearman’s ρ > 0.8, *p* < 0.05); The nodes are colored by phylum and represent an operational taxonomic unit 97% sequence identify a threshold, OTU); the size of each node is proportional to the number of connections (degrees); the thickness of each connection between two nodes (edge) is proportional to the values of Spearman’s correlation coefficient.

Co-occurrence network has been used to explore possible ecological interactions between microbial species in different environments. Therefore, we established a co-occurrence network based on the three different spatial locations of microbial taxa at the OTU level and the different drought stress-rotation patterns (W1R1-W2R1, W1R2-W2R2, W1R3-W2R3, and W1R4-W2R4) (Figure 5; Supplementary Figure S3). At the root endosphere, the W1R4−W2R4 network had the greatest number of bacteria edges, average degree, and graph density, while the W1R3-W2R3 group had the greatest network diameter (Supplementary Table S5). At the rhizosphere, the W1R3-W2R3 network had the greatest bacterial edges, average degree, and graph density (Supplementary Table S5). At the bulk soil, the W1R1-W2R1 network had the highest bacterial edges, average degree, and graph density (Supplementary Table S5). Except for W1R4-W2R4 in the rhizosphere and W1R1-W2R1 in bulk soil, the others network had typical module structure due to the calculated modularity index greater than 0.4. The W1R4-W2R4 in the endosphere, the W1R3-W2R3 in the rhizosphere, and the W1R1-W2R1 in bulk soil had the most number of bacteria-bacteria interactions.

Additionally, the proportion of positive correlations was higher than negative correlations in each microbial network (Supplementary Table S6). At the root endosphere, the W1R1-W2R1 network had the greatest number of fungi edges, average degree, and graph density, while the W1R1-W2R1 and W1R3-W2R3 groups had the greatest network diameter (Supplementary Table S5). At the rhizosphere, the greatest number of fungi edges, average degree, and graph density appeared in the W1R1−W2R1 network; the highest network diameter was in the W1R2-W2R2 group (Supplementary Table S5). At the rhizosphere, the W1R1-W2R1 network had the greatest number of fungi edges, average degree, and graph density, while the W1R2-W2R2 network had the greatest network diameter (Supplementary Table S5). Except for W1R1-W2R1 in the rhizosphere and W1R1-W2R1, W1R4-W2R4 in the rhizosphere and bulk soil, the others network had typical module structure due to the calculated modularity index being greater than 0.4. The W1R1-W2R1 had the most fungi−fungi interactions in the endosphere, rhizosphere, and bulk soil (Supplementary Table S6).

The network had a high number of abundant species. A correlation-based network analysis revealed that the key bacteria present in the endosphere of W1R1-W2R1, W1R2-W2R2, W1R3-W2R3, and W1R4-W2R4 had 1 (degree = 35), 2 (degree = 26), 7 (degree = 31), and 4 (degree = 36), respectively. One genus of W1R1−W2R2 belongs to the phylum Bacteroidota; the two genera of W1R2−W2R1 belong to the phylum Bacteroidota and Patescibacteria; the seven genera of W1R3-W2R3 belong to the phylum Cyanobacteria, Bacteroidota, Proteobacteria, and Actinobacteriota; and the four genera of W1R4-W2R4 belong to the phylum Proteobacteria and Actinobacteriota (Supplementary Table S7). The key bacteria in the rhizosphere of W1R1-W2R1, W1R2-W2R2, W1R3-W2R3, and W1R4-W2R4 had 1 (degree = 35), 2 (degree = 39), 4 (degree = 40), and 1 (degree = 39), respectively. One genus of W1R1-W2R1 belongs to the phylum Actinobacteriota; the two genera of W1R2-W2R2 belong to the phylum Actinobacteriota and Crenarchaeota; the four genera of W1R3-W2R3 belong to the phylum Actinobacteriota, Acidobacteriota, Verrucomicrobiota, and Acidobacteriota; and one genus of W1R4-W2R4 belong to the phylum Actinobacteriota (Supplementary Table S7). The key bacteria in the bulk soil of W1R1-W2R1, W1R2-W2R2, W1R3-W2R3, and W1R4-W2R4 had 13 (degree = 54), 2 (degree = 39), 10 (degree = 41) and 2 (degree = 29), respectively. Thirteen genera of W1R1-W2R1 belong to the phylum Proteobacteria, Verrucomicrobiota, Gemmatimonadota, Actinobacteriota, Acidobacteriota, and Bacteroidota; two genera of W1R2-W2R2 belong to the phylum of Firmicutes and Actinobacteriota; 10 genera of W1R3-W2R3 belong to the phylum Firmicutes, Proteobacteria, Gemmatimonadota, Actinobacteriota, and Acidobacteriota; and the two genera of W1R4-W2R4 belong to the phylum Proteobacteria (Supplementary Table S7).

Correlation-based network analysis indicated that the key fungi in the endosphere of W1R1-W2R1, W1R2-W2R2, W1R3-W2R3, and W1R4-W2R4 had 10 (degree = 36), 1 (degree = 25), 2 (degree = 26) and 1 (degree = 32), respectively. Ten genera of W1R1-W2R1 belong to the phylum Ascomycota, Basidiomycota, Chytridiomycota, k__Fungi, and Mortierellomycota; one genus of W1R2-W2R2 belong to the phylum Chytridiomycota; two genera of W1R3-W2R3 belong to the phylum Ascomycota and k__Fungi; and one genus of W1R4-W2R4 belongs to the phylum of k__Fungi. The key fungi present in the rhizosphere of W1R1-W2R1, W1R2-W2R2, W1R3-W2R3, and W1R4-W2R4 had 1 (degree = 46), 1 (degree = 29), 1 (degree = 23), and 1 (degree = 42), respectively, that belong to the phylum Mortierellomycota, Basidiomycota, and Ascomycota, respectively (Supplementary Table S8). The key fungi in the bulk soil of W1R1-W2R1, W1R2-W2R2, W1R3-W2R3, and W1R4-W2R4 had 3 (degree = 42), 3 (degree = 22), 1 (degree = 31) and 1 (degree = 27), respectively. The three genera of W1R1-W2R1 belong to the phylum Ascomycota; the three genera of W1R2-W2R2 belong to the phylum Ascomycota and Basidiomycota; one genus of W1R3-W2R3 and W1R4-W2R4 belong to the phylum Ascomycota and k__Fungi (Supplementary Table S8).

### 3.4 Relationships between soil characteristics and soil microbial community

The drought stress-rotation patterns treatment dramatically affects soil physicochemical properties, including pH, SOM, TP, TK, MBC, MBN, MBP, SC, UE, ALP, and CAT (*p* < 0.05, Supplementary Table S8). The pH value of treatments W1R1, W1R2, W1R3, W1R4, W2R2, W2R3, and W2R4 remained stable, whereas the pH value of treatment W2R1 was 8.09, which was significantly higher than other treatments. The SOM, AP, AK, MBC, MBN, and MBP of W2R1, W2R2, W2R3, and W2R4 treatments were significantly lower than the control water treatment but higher than W2R1, and the soil SC and ALP exhibited a similar trend. After drought stress, the soil UE and CAT activities in the W2R1 treatment were significantly higher than in other treatments. The comprehensive performance of spring wheat-potato-rape pattern soil was better (Table 3).

**TABLE 3:** Soil parameters and yield of different drought stress-rotation patterns.

Soil property and yield	W1R1	W1R2	W1R3	W1R4	W2R1	W2R2	W2R3	W2R4	F
pH	7.05 ± 0.07c	6.62 ± 0.02e	6.68 ± 0.07e	6.85 ± 0.04d	8.09 ± 0.07a	7.52 ± 0.1b	6.69 ± 0.03e	7.49 ± 0.09b	195.23***
SOM (g/kg)	46.24 ± 2.89b	57.33 ± 2.59a	55.93 ± 4a	55.02 ± 0.4a	43.58 ± 2.06b	54.03 ± 1.93a	53.87 ± 0.69a	53.41 ± 1.05a	13.92***
AN (g/kg)	0.35 ± 0.0052bc	0.37 ± 0.0003a	0.37 ± 0.006a	0.36 ± 0.0037b	0.34 ± 0.0007d	0.35 ± 0.0055cd	0.36 ± 0.0059b	0.35 ± 0.0045bc	13.12***
TP (g/kg)	2.85 ± 0.52ab	4.37 ± 1.29ab	5.09 ± 6.14ab	6.99 ± 5.08a	0.76 ± 0.38b	2.02 ± 0.66ab	2.19 ± 0.71ab	1.88 ± 0.32ab	1.52ns
TK (g/kg)	29.9 ± 1.49bc	33.13 ± 2.66ab	34.13 ± 3.07a	31.48 ± 1.49ab	27 ± 3.24c	32.5 ± 0.78ab	33.83 ± 0.59ab	29.87 ± 1.37bc	4.16**
MBC (mg/kg)	158.62 ± 19.61a	95.61 ± 4.58cd	105.07 ± 5.7c	129.74 ± 5.15b	45.23 ± 3.65f	95.27 ± 2.58cd	83.61 ± 1.29d	59.6 ± 2.24e	64.19***
MBN (mg/kg)	13.25 ± 2.26ab	9.45 ± 1.01de	12.29 ± 1.49abc	14.86 ± 2.15a	8.66 ± 0.93e	11.73 ± 1.28bcd	8.61 ± 0.15e	10.29 ± 1.33cde	7.17***
MBP (mg/kg)	12.09 ± 1.11cd	18.95 ± 1.65b	25.13 ± 3.8a	18.22 ± 2.73b	7.14 ± 0.35e	9.01 ± 2.19de	14.53 ± 0.4c	8.16 ± 0.65e	30.17***
SC (mg/g)	26.92 ± 0.01d	30.17 ± 0.55a	29.02 ± 0.47b	28.24 ± 0.6c	23.53 ± 0.11f	26.83 ± 0.03d	27.01 ± 0.03d	26.03 ± 0.02e	108.99***
UE (NH_4_−N mg/g)	5.33 ± 0.87b	5.73 ± 0.93b	5.72 ± 0.41b	5.67 ± 0.27b	7.22 ± 0.3a	5.81 ± 0.35b	6.06 ± 0.14b	5.95 ± 0.2b	3.56*
ALP (mg/g)	0.25 ± 0.0018c	0.27 ± 0.0053b	0.28 ± 0.0015a	0.27 ± 0.0038b	0.22 ± 0.0018d	0.26 ± 0.003c	0.27 ± 0.0045b	0.26 ± 0.0019c	97.84***
CAT (0.2mol/KMnO_4_/g)	36.17 ± 0.33c	35.5 ± 0.1d	35.44 ± 0.16d	35.61 ± 0.27d	38.11 ± 0.64a	37.15 ± 0.18b	37.12 ± 0.26b	37.74 ± 0.25a	34.34***

Note: Values for individual sites are the means of the three replicate soil cores (mean ± standard error). SOM, soil organic matter content; AN, soil available nitrogen content; TP, total phosphorus content; TK, total Soil total potassium content; MBC, soil microbial biomass carbon; MBN, soil microbial biomass nitrogen; MBP, soil microbial biomass phosphorus; SC, soil sucrase; UE, soil urease; ALP, soil alkaline phosphatase; CAT, soil catalase. Lowercase letters indicate that means of soil property are significantly different (*p* < 0.05, ANOVA) among drought stress-rotation patterns. *p < 0.05, **p < 0.01, and ***p < 0.001.

**FIGURE 6:**
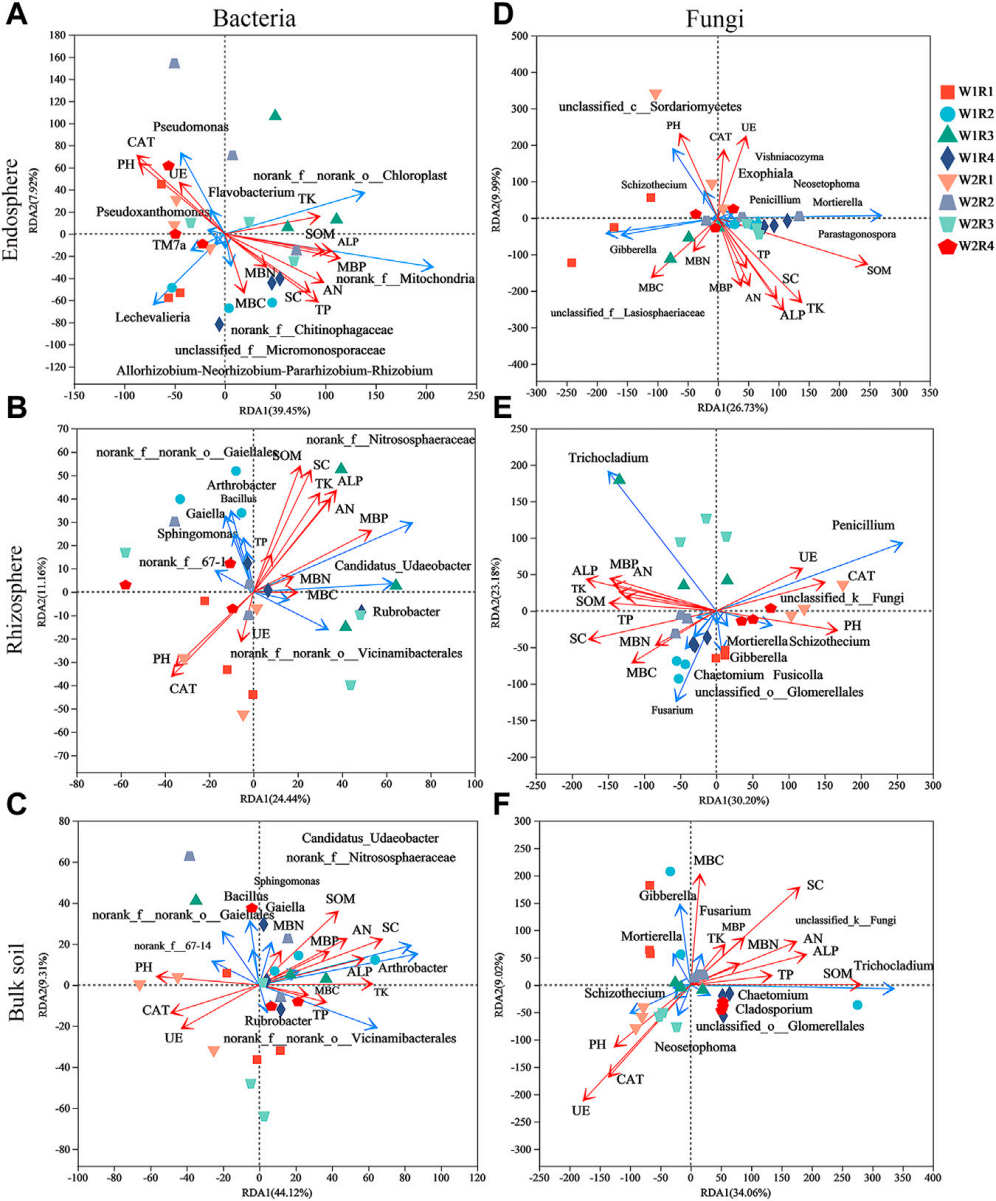
The effects of soil factors on endosphere, rhizosphere, bulk soil bacteria **(A–C)**, and fungal **(D–F)** community structure identified by Redundancy analysis (RDA). SOM, soil organic matter content; AN, soil available nitrogen content; TP, total phosphorus content; TK, total Soil total potassium content; MBC, Soil microbial biomass carbon; MBN, soil microbial biomass nitrogen; MBP, soil microbial biomass phosphorus; SC, soil sucrase; UE, Soil urease; ALP, soil alkaline phosphatase; CAT, soil catalase.

**FIGURE 7:**
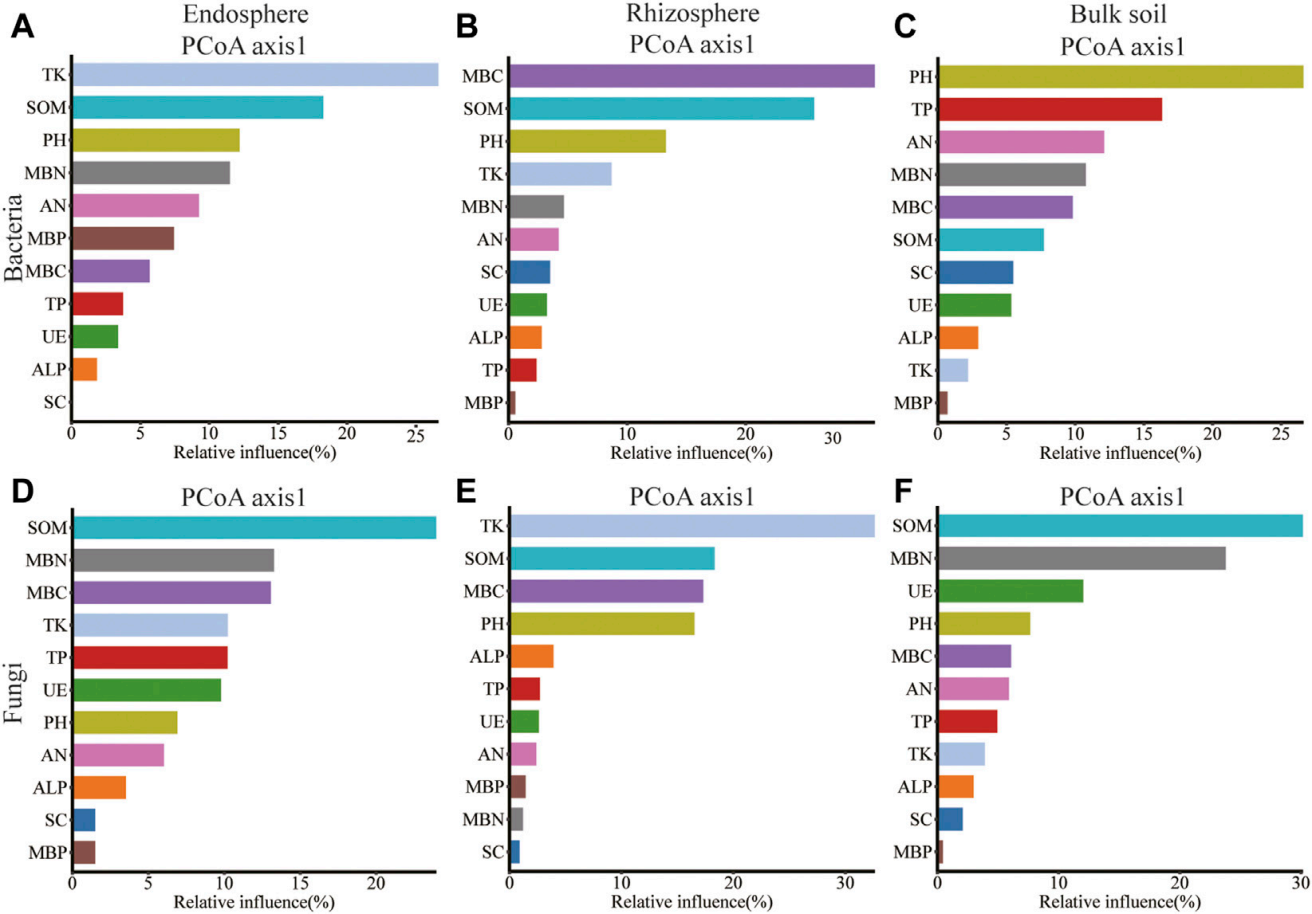
Relative variable importance plot (%) of environmental driving factors for different spatial locations composition of bacteria and fungi by ABT models, **(A–C)**: endosphere, rhizosphere, bulk soil bacteria; **(D–F)**: endosphere, rhizosphere, bulk soil fungi.

Rhizosphere *Bacillus* correlated significantly with SOM, TP, TK, SC, and ALP; *Sphingomonas* of bulk soil correlated significantly with SOM, AN, MBP, SC, and ALP; and *Rubrobacter* of rhizosphere correlated significantly with pH, MBC, MBN, and CAT (Supplementary Figures S4A–C). *Gibberella* of root endosphere correlated significantly with SOM content; *Gibberella* of rhizosphere correlated significantly with TK, MBC, and MBN content; *Penicillium* of rhizosphere correlated significantly with PH, SOM, AN, TP, MBC, MBP, SC, ALP, and CAT content; *Mortierella* of rhizosphere correlated significantly with MBC, SC, and CAT content; *Mortierella* of bulk soil correlated significantly with MBC and MBN content; *Chaetomium* of rhizosphere correlated significantly with SOM content; and *Chaetomium* of bulk soil correlated significantly with pH, MBP, SC, ALP, and CAT content (Supplementary Figures S4D–F). Spearman’s correlation test revealed that the MBC, MBP, SC, and CAT content significantly affected the alpha diversity of bacteria in the root endosphere; SOM, AN, TK, MBP, SC, ALP, and CAT content significantly affected the alpha diversity of bacteria in the rhizosphere; the SC content significantly affected the alpha diversity of bacteria in bulk soil. The MBC content significantly affected the alpha diversity of fungi in the root endosphere; the TK, MBC, and MBN content significantly affected the alpha diversity of fungi in the rhizosphere; and the SOM, SC, and UE content significantly affected the alpha diversity of fungi in bulk soil (Supplementary Table S9). The pH was related to Shannon and Simpson’s index of the rhizosphere (*p* < 0.01; Supplementary Table S9). Redundancy analysis and aggregated boosted tree exhibited that the TK and SOM affected the bacterial community structure of root endosphere; the SOM and MBC affected the bacterial community structure of rhizosphere; and pH affected the bacteria community structure of bulk soil. Meanwhile, SOM and MBN contents affected the fungal community structure of the root endosphere; TK and SOM content affected the fungal community structure of the rhizosphere; and SOM and MBN content affected the fungal community structure of bulk soil (Figures 6, 7).

### 3.5 Contrasting determinants of bacterial and fungal beta diversity

**FIGURE 8:**
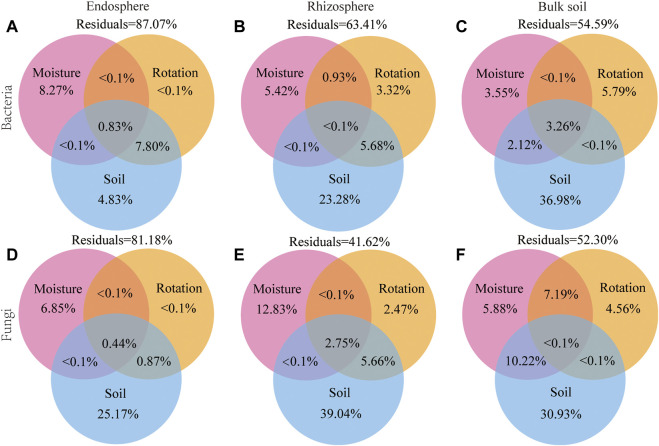
Variation partitioning analysis (VPA) of the effects of soil edaphic factors, soil moisture, and rotation patterns on endosphere, rhizosphere, and bulk soil bacterial **(A–C)** and fungal **(D–F)** communities. Soil edaphic factors include pH; SOM, soil organic matter content; AN, soil available nitrogen content; TP, total phosphorus content; TK, total Soil total potassium content; MBN, soil microbial biomass nitrogen; MBP, soil microbial biomass phosphorus; UE, Soil urease; CAT, soil catalase.

The variation partitioning analysis (VPA) confirmed that the soil factors were the driving factor for changing bacterial and fungal communities, explaining 4.83% and 25.17% of the bacteria and fungi variation in the endosphere, explaining 23.28% and 39.04% of the bacteria and fungi variation in the rhizosphere, explaining 36.98% and 30.93% of the bacteria and fungi variation in bulk soil, respectively. Differences in soil moisture and rotation patterns only explained a small percentage of the dissimilarity in microbial communities. For example, the soil moisture explains 3.55% and 5.88% of the bacterial and fungal variation in bulk soil, while the rotation patterns explain 5.79% and 4.56% of the bacterial and fungal variation in bulk soil (Figure 8). Soil edaphic factors were important predictors of microbial beta diversity (Figure 8). However, the partial Mantel test revealed that the rotation patterns also significantly correlated with the dissimilarity of soil bacterial and fungal communities (*p* < 0.01; Supplementary Table S10).

## 4 Discussion

### 4.1 Changes in microbial diversity, community structure, and co-occurrence network analysis

Our research indicates that the alpha diversity, community structure analysis of the microorganisms in the root space of spring wheat under different drought stress-rotation patterns are different, supporting our first hypothesis. Water, rotation patterns, and soil characteristics greatly affect the microbial community in the root space in drought stress-rotation patterns. Numerous studies have confirmed that drought stress influences microbial community structure (Ali and Khan, 2021; Shaffique et al., 2022) and similarly by crop rotation patterns (Paungfoo-Lonhienne et al., 2017; Liu et al., 2023). Differences in environmental factors, such as straw return, tillage practices, and other factors, can also explain changes in microbial community structure (Kraut-Cohen et al., 2019; Cong et al., 2020).

The crop rotation pattern improves soil structure, organic matter mineralization, and water-holding capacity (Mastro et al., 2019). The soil nutrient content is higher in the rotation patterns under the two water conditions, and this study’s results are consistent. Additionally, we discovered that rhizosphere soil and bulk soil have significantly higher alpha diversity change than endophytic bacteria and fungi (Supplementary Table S2), with the highest Shannon index in W1R1 and Chao1 index in W2R3 treatment among in-root bacteria, and the highest bacteria Shannon and fungal Chao1 index in W2R4 treatment among rhizosphere microorganisms. The highest bacterial and fungal Shannon indices and Chao1 indices were discovered in the W2R3 treatment in bulk soils (Supplementary Table S2), inconsistent with most studies on root space microorganisms (Bai et al., 2022; Wang et al., 2022). This is probably because rhizosphere and bulk soils under crop rotation patterns accumulate nutrients from the agricultural straw decomposition and different crop root secretions. Although agricultural ecological methods, such as crop diversification and crop rotation, can create positive legacy effects that can improve soil conditions, straw returning to the field plus tillage can break the imbalance of nutrients above and below the soil and increase the balance of soil nutrients, making the soil better or healthier, (Somenahally et al., 2018; Jing et al., 2022; Xiao et al., 2022). Rhizosphere and bulk soil alpha diversity were not significantly different but significantly higher than intra-root. Additionally, microbial alpha diversity increased when drought was encountered, probably because higher diversity and abundance of microorganisms can enhance resistance to abiotic stresses (Sandrini et al., 2022). Higher microbial diversity can also ensure stable agroecosystems and sustainable crop production (Huang et al., 2015). Therefore, crop rotation patterns produce more ecological niches to improve soil nutrient resources (Lozano et al., 2014).

PCoA and nonparametric multivariate statistical tests showed that the composition and structure of microbial communities were significantly different in spatial location, and there were significant differences under different drought stress-rotation patterns. Drought stress rotation patterns had a greater impact on fungal communities (Figure 3; Supplementary Figure S1). The abundance of major bacteria phyla root endosphere were Proteobacteria, Actinobacteriota, and Bacteroidota from high to low, while the abundance of major bacteria phyla rhizosphere and bulk soil were Actinobacteriota, Proteobacteria, and Acidobacteriota from high to low (Figure 4; Supplementary Table S6). Actinobacteriota and Acidobacteriota are important in soil carbon cycling as saprophytic bacteria can degrade inorganic carbon (Zeng et al., 2017). Our results revealed that Actinobacteriota had the highest relative abundance under W2R1 treatment within the roots and in the bulk soil. Acidobacteriota had the lowest relative abundance under W2R1 treatment in bulk soils, indicating that substrate-degrading bacteria were mainly Actinobacteriota under continuous crop treatment and Acidobacteriota under crop rotation pattern. Additionally, Proteobacteria belonged to the group of fast-growing eutrophic bacteria. Proteobacteria had the highest abundance in endosphere and rhizosphere soils in the W2R4 treatment and bulk soils in the W2R1 treatment, indicating different types of nutrient structures of microbial taxa in different spatial locations (Chao et al., 2016). The main fungal phylum in the endosphere, rhizosphere, and bulk soils was Ascomycota and Basidiomycota (Figure 5; Supplementary Table S6), and their abundance was greater in the R3 rotation pattern treatment in this study. Ascomycota primarily degrades the unstable portion of straw residue at the early stage of the decomposition process. Basidiomycota mainly decomposes refractory organic matter at the late stage of the decomposition process (D et al., 2016; Boer et al., 2005; Hollister et al., 2010). *Bacillus* has excellent resistance, growth-promoting, and biocontrol properties (Saxena et al., 2020), whereas *Sphingomonas* has important value in biodegradation, aiding phytoremediation, and enhancing plant stress resistance (Asaf et al., 2020). This study’s results indicated that the relative abundance of *Bacillus* and *Sphingomonas* in the rhizosphere and the bulk soil were higher for the rotational crop pattern than the continuous crop, and neither bacteria changed significantly in the roots (Supplementary Figure S2), indicating that *Bacillus* and *Sphingomonas* mainly functioned in the soil. Therefore, the microbial communities’ composition reflected their ecological strategies (Bi et al., 2021).

Co-occurrence network analysis demonstrates the principle of microbial community aggregation from a mathematical perspective and can describe soil microbial species’ network characteristics and interrelationships (Ma et al., 2016; Fan et al., 2017). In our study, the co-occurrence network of soil microorganisms exhibited less negative correlation in the root space under different crop rotation patterns (Supplementary Table S6). This may be due to the abundance of crop root space resources, reducing competition among microorganisms and allowing more species to maintain free-living populations (Costello et al., 2012). Moreover, root endosphere, rhizosphere, and bulk soil bacteria interactions were stable under the different rotations, whereas fungi interactions were higher in the root endosphere, rhizosphere, and bulk soil of wheat in the continuous crop pattern than in the rotational crop, indicating that the continuous crop pattern microorganisms need more interactions to enhance its adaptability to the environment. Therefore, crop rotation microbial co-occurrence network becomes more robust and stable than continuous cropping (Yang et al., 2023). The W1R3-W2R3 network had more bacterial and fungal critical species in the rhizosphere and bulk soils (Figure 5; Supplementary Figure S3; Supplementary Tables S7, S8). These different microbial distribution patterns may be related to the assembly process of microbial communities under different crop rotation patterns.

### 4.2 Drivers of microbial community variation

In arid regions, soil moisture is an important factor governing vegetation growth (Jia et al., 2017). The soil moisture significantly affects changes in microbial community structure on the Loess Plateau during 30 years of grassland restoration (Guo et al., 2019). Shi Gongfu et al. demonstrated that crop rotation fallow patterns significantly affected root space microbial diversity, species abundance, and community structure (Shi, 2021). Although soil moisture and crop rotation pattern treatment involve vegetation growth in arid areas, it is not a driving factor for changes in soil microbial communities under drought stress-rotation pattern treatment.

Our results revealed that the SOM and MBC affected the bacteria community structure of the rhizosphere, while the pH affected the bacteria community structure of bulk soil. Meanwhile, the SOM and MBN contents affected the fungal community structure of the root endosphere. The TK and SOM content affected the fungal community structure of the rhizosphere, while the SOM and MBN content affected the fungal community structure of bulk soil (Figures 6, 7). Microorganisms play an important role in the formation and decomposition of SOM, and soil aggregates provide a habitat for microorganisms and physical protection for organic matter (Angst et al., 2021). Soil pH is a key regulator of microbial community distribution, affecting soil microbial community dynamics and ecological processes during plant growth (Tripathi et al., 2018). Soil microbial biomass participates in the nutrient cycling process of the soil system and is one of the key links between plants and soil nutrients. It is a repository of active soil nutrients and a source and reservoir of nutrients required for plant growth and development (Mcdaniel and Grandy, 2016; Singh and Gupta, 2018). Straw returned to the field, and potassium fertilizer application may increase potassium’s sensitivity to factors affecting the spatial community composition of bacteria and fungi. This hypothesis will be further verified.

### 4.3 Significance of soil microbial community variation under drought stress-rotation patterns treatment

The different responses of bacterial and fungal communities to environmental factors described in our study provided further evidence of the diversity of microbiota functions. Beneficial bacteria, such as *Bacillus* and *Sphingomonas*, facilitate straw decomposition, nutrient conversion, and accumulation as crop rotation increases the straw decomposition rate yearly. Therefore, soil microorganisms have community-building mechanisms in the dry crop area and spatial distribution characteristics in the western foothills of the Greater Khingan Range. The distribution characteristics are triple influenced by spatial location, environmental factors, and crop rotation patterns, presenting an apparent spatial distribution pattern of microorganisms. Different microbial taxa can play different metabolic functions in the dry farming ecosystem and play a resilient role under specific environmental conditions.

## 5 Conclusion

The results exhibited significant differences in the microbial community structure at different spatial locations under the drought stress-rotation patterns. Different drought stress-rotation patterns had a greater impact on the fungal community. The topological characteristics of the co-occurrence network indicate that the bacteria community is relatively stable without significant changes in each rotation pattern. However, the fungi co-occurrence network is more stable in the crop rotation patterns. The fungal community is less stable in the continuous cropping pattern; species respond to drought through enhanced interactions. Soil factors were the direct factors influencing the changes in microbial communities at different locations. Among them, SOM, MBN and pH were the most important factors dominating the changes in bacterial community structure in the endosphere, rhizosphere and bulk soil, respectively. The dominant factor affecting changes in fungal community structure in the endosphere, rhizosphere and bulk soil was SOM. Crop rotation patterns and soil moisture were the indirect factors affecting changes in microbial community structure.

## Data Availability

The datasets presented in this study can be found in online repositories. The names of the repository/repositories and accession number(s) can be found below: https://www.ncbi.nlm.nih.gov/, PRJNA943477.
