# Supplementary material for: Dynamic changes of soil microorganisms in rotation farmland at the western foot of the Greater Khingan range
Source: Front Bioeng Biotechnol. 2023 Jun 23;11:1191240. doi: 10.3389/fbioe.2023.1191240 (PMC10328388; doi:10.3389/fbioe.2023.1191240)
Supplement: Supplementary file 1 [file DataSheet1.pdf]

Supplementary Material

Dynamic changes of soil microorganisms in rotation farmland at the western foot of the Greater Khingan Range

Shuli Wei[1,2,3,4], Jing Fang[1,3,4], Tianjiao Zhang[1,3,4], Jianguo Wang[2,3,4], Yuchen Cheng[2,3,4], Jie Ma[1,3,4], Rui Xie[2,3,4], Zhixiong Liu[2], Erhu Su[2], Yongfeng Ren[1,2,3,4], Xiaoqing Zhao[1,2,3,4*], Xiangqian Zhang[1,2,3,4*], Zhanyuan Lu[1,2,3,4*]

[1] School of Life Science, Inner Mongolia University, Huhhot, China

[2] X Inner Mongolia Academy of Agricultural & Animal Husbandry Sciences, Huhhot, China

[3] Key Laboratory of Black Soil Protection And Utilization (Hohhot), Ministry of Agriculture and Rural Affairs, P.R.China

[4] Inner Mongolia Key Laboratory of Degradation Farmland Ecological Remediation and Pollution Control, Huhhot, China.

*** Correspondence:**
Xiaoqing Zhao
zhaoxq204@163.com

Xiangqian Zhang
zhangxiangqian_2008@126.com

Zhanyuan Lu
lzhy2811@163.com

1 Supplementary Tables

Supplementary Table S1 Sequence number for each sample.

Sample ID	Endosphere		Rhizosphere		Bulk soil	
	**Bacteria**	**Fungi**	**Bacteria**	**Fungi**	**Bacteria**	**Fungi**
	Valid sequence	Valid sequence	Valid sequence	Valid sequence	Valid sequence	Valid sequence
W1R1-1	141590	161055	140487	119436	198142	115545
W1R1-2	124483	166160	132153	123494	141056	108697
W1R1-3	105093	166229	129170	112166	154045	106987
W1R2-1	117802	127940	134169	128767	129206	140327
W1R2-2	144331	108394	139055	117155	159385	129907
W1R2-3	119720	119793	121178	132668	154164	145712
W1R3-1	107557	110289	165455	121142	142607	127206
W1R3-2	120810	119049	174133	125766	171235	118149

W1R3-3	116104	140114	205946	145058	145335	122071
W1R4-1	112606	116269	129177	117433	143714	133592
W1R4-2	116886	108904	142261	129510	163239	154156
W1R4-3	102407	110456	145742	130673	183984	128869
W2R1-1	185029	112690	145453	140237	125334	172965
W2R1-2	140690	131440	81879	127680	118687	124563
W2R1-3	161004	110552	101913	114748	94061	130362
W2R2-1	108799	151025	112266	132111	164015	128288
W2R2-2	130719	148745	135311	163429	294396	143944
W2R2-3	75282	115326	140514	124938	265163	152390
W2R3-1	139385	121531	207706	144736	131434	126967
W2R3-2	219209	132458	173198	136324	128111	125562
W2R3-3	140984	133957	88241	147103	139306	126463
W2R4-1	132582	138800	145979	131792	209625	125220
W2R4-2	114603	99290	255124	141675	151396	151196
W2R4-3	105349	118633	213826	133467	167823	127855
Total	3083024	3069099	3560336	3141508	3875463	3166993

Supplementary Table S2 Differences in bacterial and fungal alpha diversity among the soil of the treatment of different drought stress−rotation patterns

Root spatial	Alpha diversity	Kingdoms	W1R1	W1R2	W1R3	W1R4	W2R1	W2R2	W2R3	W2R4	($F_{W1R1, W2R4}$)
Endosphere	Observed OTUs	bacteria	2496±187.02a(B)	2615.67±508.83a (B)	2375.33±216.26a (B)	2323.33±123.03a (B)	2316±16.09a(B)	2240±242.28a (B)	2668±429.65a(B)	1698.67±227.45b (B)	3.32*
		Fungi	519±70.93a(B)	395±36.37bc(B)	384±39.74bc(B)	408±37.24b(C)	393±5bc(C)	428.33±55.08b (B)	451±47.57ab(C)	321±9.17c(C)	5.35**
	Shannon index	bacteria	5.13±0.14ab(B)	5.02±0.15abc(B)	4.89±0.28abc(B)	4.78±0.07c(B)	4.92±0.13abc (B)	4.85±0.15bc(B)	5.2±0.25a(B)	4.9±0.13abc(B)	1.99
		Fungi	3.22±0.92a(B)	3.51±0.11a(A)	3.43±0.41a(B)	3.02±0.12a(C)	3.23±0.46a(C)	3.19±0.53a(B)	3.28±0.23a(B)	3±0.24a(C)	0.46
	Chao1 estimator	bacteria	3138.72±61.25a(B)	3153.12±403a (B)	2898.86±297.11a (B)	2969.97±262.6a (B)	2649.22±53.72a (B)	2888.11±185.5a (B)	3033.46±399.95a (B)	2166.55±198.41b (B)	4.49**
		Fungi	582.5±103.23a(B)	427.89±23.11bc (B)	423.91±49.81bc (B)	467.66±44.96b (C)	456.48±16.44b (B)	468.97±44.62b (B)	497.22±60.53ab (C)	352.82±11.14c (C)	4.79**
Rhizosphere	Observed OTUs	bacteria	5214.33±113.37ab(A)	4586±77.54b(A)	5507±431.8ab(A)	5012±57.16ab(A)	4913.33±481.67ab (A)	4973.67±206.03ab (A)	4811±918.86ab (A)	5730.67±834.83a (A)	1.66
		Fungi	1300.67±143.11a (A)	1135.33±27.5b(A)	1047.33±50.86b(A)	1296.33±36.46a (A)	1139.67±1.15b(B)	1086±52.31b(A)	1020±61.1b(B)	1343.67±35.23a (A)	11.47***
	Shannon index	bacteria	6.61±0.06ab(A)	6.31±0.1d(A)	6.38±0.16cd(A)	6.48±0.08bc(A)	6.68±0.1a(A)	6.54±0.05abc (A)	6.42±0.03cd(A)	6.62±0.06ab(A)	6.50**
		Fungi	4.9±0.1a(A)	4.43±0.11bc (AB)	3.89±0.56d(A)	4.87±0.04a(A)	4.08±0.29cd(B)	3.99±0.09d(A)	3.8±0.13d(B)	4.58±0.05ab(A)	10.07***
	Chao1 estimator	bacteria	6014.41±208.75ab (A)	5635.22±162.12b (A)	5910.2±252.59ab (A)	5841.74±104.11ab (A)	5982.98±375.36ab (A)	5951.85±217.33ab (A)	5553.61±488.93b (A)	6274.02±518.82a (A)	1.45ns
		Fungi	1514.36±196.74a (A)	1300.51±50.32bc (A)	1224.83±73.04c (A)	1463.4±41.59ab (A)	1326.47±46.84bc (A)	1213.69±41.11c (A)	1221.06±77.31c (B)	1519.04±84.43a (A)	6.42**
Bulk soil	Observed OTUs	bacteria	5386±254.03ab(A)	4889±173.23bc (A)	5141.67±321.5abc (A)	5190±274.87abc (A)	4556±444.03bc (A)	5951±841.09a (A)	5282.33±431.21abc (A)	5168±252.53abc (A)	2.69*
		Fungi	1099±38.04abc(A)	1112±149.4c(A)	1008±116.98abc (A)	1076±89.35bc (B)	1350±132.14ab (A)	1119±23.35bc (A)	1358±206.66a(A)	1082±5.66c(B)	2.67*
	Shannon index	bacteria	6.53±0.01ab(A)	6.31±0.09c(A)	6.46±0.04bc(A)	6.51±0.08ab(A)	6.59±0.08ab(A)	6.44±0.07bc(A)	6.65±0.2a(A)	6.45±0.03bc(A)	3.76*
		Fungi	4.74±0.18ab(A)	3.71±1.06c(A)	4.59±0.22ab(A)	4.03±0.18bc(B)	4.94±0.21a(A)	4.13±0.19abc (A)	4.66±0.48ab(A)	4.23±0.1abc(B)	2.74*
	Chao1 estimator	bacteria	6232.97±122.1abc (A)	5845.61±182.47bc (A)	6084.06±120.08abc (A)	5983.31±92.87abc (A)	5714.6±525.34c (A)	6289.43±284.29ab (A)	6430.73±426.72a(A)	5766.49±187.69bc (A)	2.53
		Fungi	1268.88±98.58ab (A)	1184.11±137.36b (A)	1280.3±153.16ab (A)	1241.46±108.4b (B)	1376±17.01ab (A)	1198.69±74.9b (A)	1484.28±203.85a (A)	1191.68±33.75b (B)	2.32

Note: All data are presented as the mean ± standard error. Lowercase letters indicate that the means of alpha diversity index are significantly different ($P < 0.05$) among different drought stress−rotation patterns, capital indicate that the means of alpha diversity index are significantly different ($P < 0.05$) among spatial location of different wheat roots under the same drought stress−rotation treatment. *$P < 0.05$, **$P < 0.01$, and ***$P < 0.001$.

Supplementary Table S3 Relative abundances of the main soil bacteria phyla of different drought stress−rotation patterns treatment

Root spatial	Phyum of phyum	W1R1	W1R2	W1R3	W1R4	W2R1	W2R2	W2R3	W2R4
Endosphere	Actinobacteriota	0.2106	0.2139	0.1102	0.2249	0.2354	0.139	0.1894	0.1302
	Proteobacteria	0.5151	0.4343	0.4499	0.452	0.4762	0.5339	0.4682	0.5504
	Acidobacteriota	0.0065	0.0157	0.0157	0.0107	0.0084	0.0143	0.0095	0.004
	Chloroflexi	0.0165	0.022	0.0434	0.0259	0.0145	0.0301	0.0436	0.0105
	Crenarchaeota	/	/	/	/	/	/	/	/
	Verrucomicrobiota	0.007	0.017	0.0254	0.0091	0.007	0.0148	0.0179	0.0112
	Firmicutes	/	/	/	/	/	/	/	/
	Bacteroidota	0.1665	0.1605	0.1441	0.0987	0.1269	0.1418	0.1499	0.1821
	Planctomycetota	0.0037	0.011	0.0143	0.0066	0.0048	0.0059	0.0066	0.0024
	Gemmatimonadota	/	/	/	/	/	/	/	/
	Myxococcota	0.0146	0.0092	0.0237	0.0078	0.016	0.0185	0.0181	0.0136
	Cyanobacteria	0.0147	0.054	0.1482	0.0665	0.0318	0.0566	0.0653	0.0232
	Patescibacteria	0.0312	0.048	0.0102	0.0844	0.0709	0.0329	0.0137	0.0639
Rhizosphere	Actinobacteriota	0.3078	0.3248	0.2535	0.2686	0.3155	0.3122	0.2898	0.291
	Proteobacteria	0.2161	0.2298	0.1481	0.2061	0.2098	0.2205	0.191	0.2749
	Acidobacteriota	0.1139	0.091	0.1285	0.1305	0.1093	0.0996	0.1214	0.0782
	Chloroflexi	0.0799	0.0621	0.0912	0.0607	0.0692	0.0602	0.0818	0.0558
	Crenarchaeota	0.046	0.063	0.1194	0.0776	0.0695	0.0623	0.0605	0.062
	Verrucomicrobiota	0.0459	0.036	0.0672	0.0637	0.0295	0.0345	0.0614	0.0246
	Firmicutes	0.0345	0.0547	0.0392	0.0425	0.0315	0.0548	0.0439	0.0408
	Bacteroidota	0.0408	0.0417	0.0306	0.0382	0.048	0.0418	0.0374	0.0625
	Planctomycetota	0.0373	0.0202	0.0472	0.0385	0.0303	0.0211	0.0404	0.0189
	Gemmatimonadota	0.0281	0.0366	0.0264	0.0306	0.0248	0.0352	0.0294	0.031
	Myxococcota	0.0181	0.0151	0.0164	0.0156	0.0255	0.0239	0.0154	0.0305
	Cyanobacteria	/	/	/	/	/	/	/	/
	Patescibacteria	/	/	/	/	/	/	/	/
Bulk soil	Actinobacteriota	0.3205	0.277	0.2927	0.2873	0.3588	0.3101	0.2721	0.287
	Proteobacteria	0.2075	0.1792	0.2096	0.2082	0.2699	0.211	0.2163	0.2047
	Acidobacteriota	0.1181	0.129	0.1062	0.1078	0.063	0.0919	0.1231	0.1111
	Chloroflexi	0.0644	0.0618	0.0601	0.0679	0.0548	0.0612	0.0725	0.0661
	Crenarchaeota	0.057	0.0933	0.0829	0.0733	0.0384	0.0872	0.0662	0.0681
	Verrucomicrobiota	0.0453	0.0692	0.0464	0.0514	0.014	0.0302	0.0406	0.0555
	Firmicutes	0.0447	0.0474	0.0508	0.0476	0.0445	0.0578	0.0414	0.0536
	Bacteroidota	0.0316	0.0328	0.0392	0.0404	0.0509	0.0386	0.0454	0.0415
	Planctomycetota	0.0321	0.0361	0.0293	0.0355	0.0167	0.018	0.0398	0.0332
	Gemmatimonadota	0.0311	0.0325	0.0338	0.0345	0.0335	0.0369	0.0303	0.0364
	Myxococcota	0.0172	0.0149	0.0194	0.0184	0.0266	0.0242	0.0231	0.0161
	Cyanobacteria	/	/	/	/	/	/	/	/
	Patescibacteria	/	/	/	/	/	/	/	/

Supplementary Table S4 Relative abundances of the main soil fungi phyla of different drought stress−rotation patterns treatment

Root spatial	Phyum of fungi	W1R1	W1R2	W1R3	W1R4	W2R1	W2R2	W2R3	W2R4
Endosphere	Ascomycota	0.8791	0.7151	0.6433	0.7838	0.8453	0.7461	0.7382	0.8782
	Mortierellomycota	0.037	0.062	0.0505	0.0486	0.0441	0.0704	0.0447	0.0945
	Basidiomycota	0.063	0.2021	0.2901	0.1457	0.0794	0.123	0.1984	0.0195
	k__Fungi	0.0165	0.0098	0.0115	0.0091	0.0057	0.0079	0.0032	0.0037
	Chytridiomycota	0.0019	0.0054	0.0009	0.0087	0.0185	0.0006	0.0112	0.0001
	Olpidiomycota	0.0003	0.003	0.0005	0.0029	0.0055	0.0513	0.0037	0.0033
Rhizosphere	Ascomycota	0.6618	0.7523	0.7259	0.741	0.7786	0.8442	0.7713	0.7295
	Mortierellomycota	0.1662	0.1289	0.1324	0.1225	0.1053	0.1079	0.1058	0.0769
	Basidiomycota	0.1074	0.0748	0.1001	0.0888	0.0555	0.0217	0.0953	0.1483
	k__Fungi	0.0372	0.025	0.0185	0.0252	0.0233	0.0139	0.0157	0.0173
	Chytridiomycota	0.016	0.0129	0.0082	0.0158	0.0131	0.0056	0.0075	0.0149
	Olpidiomycota	0.0014	0.0014	0.0018	0.0021	0.0135	0.0019	0.0009	0.0084
Bulk soil	Ascomycota	0.6512	0.8239	0.7688	0.749	0.7337	0.8297	0.7643	0.7486
	Mortierellomycota	0.1829	0.0986	0.1294	0.1165	0.0925	0.1098	0.0873	0.1272
	Basidiomycota	0.1018	0.0438	0.0521	0.0816	0.0808	0.0273	0.0977	0.0739
	k__Fungi	0.0356	0.0187	0.0258	0.0299	0.042	0.0194	0.0217	0.0235
	Chytridiomycota	0.0198	0.0097	0.0143	0.0094	0.0365	0.0063	0.0185	0.021
	Olpidiomycota	0.0013	0.0015	0.0021	0.0018	0.0061	0.0021	0.0065	0.0023

Supplementary Table S5 Topological features of co−occurrence network of soil microbial communities of different drought stress−rotation patterns treatment (corresponding to Figure 5, Figure S3)

Toplogical features	Endospherere				Rhizosphere				Bulk soil			
	**W1R1 – W2R1**	**W1R2 – W2R2**	**W1R3 – W2R3**	**W1R4 – W2R4**	**W1R1 – W1R2**	**W1R2 – W2R2**	**W1R3 – W2R3**	**W1R4 – W2R4**	**W1R1 – W2R1**	**W1R2 – W2R2**	**W1R3 – W2R3**	**W1R4 – W2R4**
**Bacteria Network metrics**												
Nodes	98	99	99	98	100	98	99	98	100	97	98	99
Edges	766	604	652	889	756	858	1091	905	1495	872	1046	630
Modularity	0.417	0.489	0.417	0.404	0.450	0.441	0.425	0.367	0.144	0.403	0.431	0.403
Network diameter	7	6	9	6	9	5	7	7	10	6	9	7
Average degree	15.633	12.202	13.172	18.143	15.12	17.51	22.04	18.469	29.18	17.979	21.347	12.727
Graph density	0.161	0.125	0.134	0.187	0.153	0.181	0.225	0.180	0.295	0.187	0.220	0.130
Average path length	2.674	2.731	3.251	2.467	2.826	2.493	2.681	2.676	2.549	2.533	2.720	2.910
Average clustering coeffcient	0.629	0.634	0.626	0.606	0.632	0.623	0.685	0.675	0.722	0.62	0.712	0.547
**Fungi Network metrics**												
Nodes	97	100	97	96	97	99	98	98	98	99	98	99
Edges	872	507	545	621	1242	642	540	1028	1145	542	565	503
Modularity	0.336	0.476	0.455	0.445	0.317	0.406	0.494	0.298	0.269	0.505	0.409	0.52
Network diameter	9	7	9	7	7	11	8	9	6	6	10	10
Average degree	17.979	10.14	11.237	12.938	25.608	12.97	11.02	20.98	23.367	10.949	11.531	10.162
Graph density	0.187	0.102	0.117	0.136	0.267	0.132	0.114	0.216	0.241	0.112	0.119	0.104
Average path length	2.686	3.03	3.211	2.915	2.075	3.367	3.014	2.583	2.203	2.928	3.052	3.317
Average clustering coeffcient	0.62	0.516	0.593	0.557	0.616	0.486	0.545	0.595	0.625	0.53	0.535	0.548

Supplementary Table S6 Topological features of co−occurrence network of soil microbial communities of different drought stress−rotation patterns treatment (corresponding to Figure 5, Figure S3)

Group	Taxa	Correlation	Endosphere		Rhizosphere		Bulk soil	
			Bacteria	Fungi	Bacteria	Fungi	Bacteria	Fungi
W1R1−W2R1	Bacteria	Positive	446(58.22%)	−	390(51.59%)	−	855(58.60%)	−
		Negative	320(41.78%)	−	366(48.41%)	−	604(41.40%)	−
	Fungi	Positive	−	502(57.57%)	−	736(59.26%)	−	594(51.88%)
		Negative	−	370(42.43%)	−	506(40.74%)	−	551(48.12%)
W1R2−W2R2	Bacteria	Positive	372(61.59%)	−	568(66.20%)	−	562(64.45%)	−
		Negative	232(38.41%)	−	290(33.80%)	−	310(35.55%)	−
	Fungi	Positive	−	263(51.87%)	−	381(59.35%)	−	450(83.03%)
		Negative	−	244(48.13%)	−	261(40.65%)	−	92(16.97%)
W1R3−W2R3	Bacteria	Positive	432(66.26%)	−	638(58.48%)	−	700(66.92%)	−
		Negative	220(33.74%)	−	453(41.52%)	−	346(33.08%)	−
	Fungi	Positive	−	311(57.06%)	−	366(67.78%)	−	310(54.86%)
		Negative	−	234(42.94%)	−	174(32.22%)	−	255(45.13%)
W1R4−W2R4	Bacteria	Positive	525(59.06%)	−	576(63.65)	−	349(55.40%)	−
		Negative	364(40.94%)	−	329(36.35)	−	281(44.60%)	−
	Fungi	Positive	−	370(59.58%)	−	558(52.28%)	−	305(60.64%)
		Negative	−	251(40.42%)	−	470(45.72%)	−	198(39.36%)

Supplementary Table S7 The highest degree in different bacteria network diagrams under drought stress-rotation patterns (corresponding to Figure 5).

Root spatial	Group	Degree	OTU	Genus	Phylum
Endospherere	W1R1-W2R1	35	OTU8415	f__Microscillaceae	Bacteroidota
	W1R2-W2R2	26	OTU14109	f__Chitinophagaceae	Bacteroidota
		26	OTU8138	f__LWQ8	Patescibacteria
	W1R3-W2R3	31	OTU8101	f__norank	Cyanobacteria
		31	OTU7189	f__Chitinophagaceae	Bacteroidota
		31	OTU13624	f__Comamonadaceae	Proteobacteria
		31	OTU12587	Kribbella	Actinobacteriota
		31	OTU11144	Nocardioides	Actinobacteriota
		31	OTU8053	f__Comamonadaceae	Proteobacteria
		31	OTU8225	o__Chloroplast	Cyanobacteria
	W1R4-W2R4	36	OTU12260	Luteimonas	Proteobacteria
		36	OTU12415	Polaromonas	Proteobacteria
		36	OTU12506	Lysobacter	Proteobacteria
		36	OTU13272	f__Intrasporangiaceae	Actinobacteriota
Rhizosphere	W1R1-W1R2	35	OTU16065	o__Gaiellales	Actinobacteriota
	W1R2-W2R2	39	OTU12406	Massilia	Actinobacteriota
		39	OTU12763	Gaiella	Crenarchaeota
	W1R3-W2R3	40	OTU12269	Blastococcus	Actinobacteriota
		40	OTU11615	o__Vicinamibacterales	Acidobacteriota
		40	OTU13267	Candidatus_Udaeobacter	Verrucomicrobiota
		40	OTU11023	Bryobacter	Acidobacteriota
	W1R4-W2R4	39	OTU10965	Gaiella	Actinobacteriota
Bulk soil	W1R1-W1R2	54	OTU12406	Massilia	Proteobacteria
		54	OTU13295	Candidatus_Udaeobacter	Verrucomicrobiota
		54	OTU14249	f__Gemmatimonadaceae	Gemmatimonadota
		54	OTU13061	Solirubrobacter	Actinobacteriota
		54	OTU11704	Microvirga	Proteobacteria
		54	OTU12564	o__Vicinamibacterales	Acidobacteriota
		54	OTU11548	Candidatus_Udaeobacter	Verrucomicrobiota
		54	OTU13331	RB41	Acidobacteriota
		54	OTU12188	Rubrobacter	Actinobacteriota
		54	OTU11303	Agromyces	Actinobacteriota
		54	OTU11900	Solirubrobacter	Actinobacteriota
		54	OTU11169	Streptosporangium	Actinobacteriota
		54	OTU11402	Terrimonas	Bacteroidota
	W1R2-W2R2	39	OTU4540	Bacillus	Firmicutes
		39	OTU12238	Gaiella	Actinobacteriota
	W1R3-W2R3	41	OTU13635	Bacillus	Firmicutes
		41	OTU12898	Sphingomonas	Proteobacteria
		41	OTU12950	Bradyrhizobium	Proteobacteria
		41	OTU14249	f__Gemmatimonadaceae	Gemmatimonadota
		41	OTU4729	f__67-14	Actinobacteriota
		41	OTU11202	f__67-14	Actinobacteriota
		41	OTU14058	Pseudolabrys	Proteobacteria
		41	OTU12253	f__Vicinamibacteraceae	Acidobacteriota
		41	OTU12956	o__Gaiellales	Actinobacteriota
		41	OTU10939	o__Gaiellales	Actinobacteriota
	W1R4-W2R4	29	OTU11602	c__Gammaproteobacteria	Proteobacteria
		29	OTU11233	f__SC-I-84	Proteobacteria

Supplementary Table S8 The highest degree in different fungi network diagrams under drought stress-rotation patterns (corresponding to Figure S3).

Root spatial	Group	Degree	OTU	Genus	Phylum
Endospherere	W1R1-W2R2	36	OTU3175	o__Sordariales	Ascomycota
		36	OTU4227	Schizothecium	Ascomycota
		36	OTU3628	Calyptella	Basidiomycota
		36	OTU3231	p__Chytridiomycota	Chytridiomycota
		36	OTU796	Penicillium	Ascomycota
		36	OTU857	k__Fungi	k__Fungi
		36	OTU3720	f__Orbiliaceae	Ascomycota
		36	OTU1055	Coprinellus	Ascomycota
		36	OTU3645	Mortierella	Mortierellomycota
		36	OTU1134	Coniochaeta	Ascomycota
	W1R2-W2R2	25	OTU3860	p__Chytridiomycota	Chytridiomycota
	W1R3-W2R3	26	OTU3659	Fusicolla	Ascomycota
		26	OTU3709	k__Fungi	k__Fungi
	W1R4-W2R4	32	OTU1130	k__Fungi	k__Fungi
Rhizosphere	W1R1-W2R1	46	OTU3645	Mortierella	Mortierellomycota
	W1R2-W2R2	29	OTU3008	Cutaneotrichosporon	Basidiomycota
	W1R3-W2R3	23	OTU3562	Conocybe	Basidiomycota
	W1R4-W2R4	42	OTU3546	Thelebolus	Ascomycota
Bulk soil	W1R1-W1R2	42	OTU3906	f__Lasiosphaeriaceae	Ascomycota
		42	OTU3688	Fusarium	Ascomycota
		42	OTU3499	Pleotrichocladium	Ascomycota
	W1R2-W2R2	22	OTU3889	Gibberella	Ascomycota
		22	OTU3698	Parastagonospora	Ascomycota
		22	OTU3008	Cutaneotrichosporon	Basidiomycota
	W1R3-W2R3	31	OTU3533	o__Sordariales	Ascomycota
	W1R4-W2R4	27	OTU556	k__Fungi	k__Fungi

Supplementary Table S9 Spearman's correlation between bacterial and fungal alpha diversity of dfferent spatial location sand soil variables.

Root spatial	Kingdoms	Alpha diversity	PH	SOM	AN	TP	TK	MBC	MBN	MBP	SC	UE	ALP	CAT
Endosphere	Bacteria	Observed OTUs	**−0.55****	0.19	0.28	0.22	0.40	**0.55****	0.09	**0.53****	**0.52****	−0.30	0.36	**−0.53****
		Shannon Index	−0.23	−0.06	−0.18	−0.33	0.25	0.10	−0.28	−0.10	0.04	−0.13	0.01	0.04
		Chao1 estimator	**−0.50***	0.14	0.17	0.15	**0.48***	**0.50***	0.14	0.37	**0.46***	−0.38	0.28	**−0.53****
	Fungi	Observed OTUs	−0.25	−0.10	−0.19	0.05	0.00	**0.53****	0.19	0.00	0.10	−0.25	0.03	−0.20
		Shannon Index	−0.21	0.15	0.16	−0.11	0.30	0.05	−0.18	0.19	0.22	−0.10	0.10	−0.10
		Chao1 estimator	−0.13	−0.30	−0.25	0.00	−0.05	**0.53****	0.17	−0.05	−0.03	−0.27	−0.08	−0.09
Rhizosphere	Bacteria	Observed OTUs	**0.56****	**−0.49***	**−0.47***	−0.08	**−0.51***	−0.05	0.23	**−0.52 ****	**−0.51***	0.07	**−0.52 ****	0.32
		Shannon Index	**0.75****	**−0.70 ****	**−0.66 ****	−0.10	**−0.65 ****	−0.21	0.00	**−0.63 ****	**−0.77****	0.35	**−.073****	**0.63****
		Chao1 estimator	**0.42***	−0.26	−0.26	−0.03	−0.30	−0.09	0.21	−0.28	−0.30	0.17	−0.30	0.21
	Fungi	Observed OTUs	0.16	−0.15	−0.17	0.02	**−0.46***	0.32	0.35	−0.25	−0.10	−0.25	−0.24	−0.01
		Shannon Index	−0.04	−0.08	−0.11	0.03	−0.15	**0.48***	**0.41***	−0.09	0.13	−0.31	−0.06	−0.21
		Chao1 estimator	0.14	−0.24	−0.15	−0.01	**−0.42***	0.19	0.25	−0.21	−0.16	−0.27	−0.22	−0.02
Bulk soil	Bacteria	Observed OTUs	−0.17	−0.02	−0.22	−0.07	0.07	0.18	0.07	−0.02	−0.07	−0.01	0.07	−0.01
		Shannon Index	0.19	−0.40	−0.29	−0.08	−0.20	−0.10	−0.06	−0.21	**−0.50***	0.29	−0.27	0.31
		Chao1 estimator	−0.28	0.08	−0.02	0.06	0.39	0.33	0.21	0.04	0.14	−0.22	0.26	−0.16
	Fungi	Observed OTUs	0.09	−0.29	−0.24	−0.33	0.00	−0.16	−0.35	−0.02	−0.40	0.24	−0.23	0.27
		Shannon Index	0.28	**−0.62 ****	−0.31	−0.24	−0.22	−0.08	−0.16	−0.16	**−0.43***	**0.43***	−0.36	0.33
		Chao1 estimator	0.01	−0.19	−0.11	−0.06	−0.10	−0.11	−0.25	0.05	−0.25	**0.50***	−0.10	0.25

Note: SOM, soil organic matter content; AN, soil available nitrogen content; TP, total phosphorus content; TK, total Soil total potassium content; MBC, Soil microbial biomass carbon; MBN, soil microbial biomass nitrogen; MBP, soil microbial biomass phosphorus; SC, soil sucrase; UE, Soil urease; ALP, soil alkaline phosphatase; CAT, soil catalase; * $P < 0.05$, ** $P < 0.01$, and ***$P < 0.001$.

Supplementary Table S10 Relationships among dissimilarities of the bacterial and fungal community composition of different spatial locations, soil moisture, rotations and soil edaphic factors identified by partial Mantel tests.

Taxon	Root spatial	Variables	Control for	Mantel R	P
Bacteria	Endosphere	Moisture	Rotation；Soil factors	0.034	0.284
		Rotation	Moisture；Soil factors	0.091	0.131
		Soil factors	Moisture；Rotation	0.266	0.001**
	Rhizosphere	Moisture	Rotation；Soil factors	0.142	0.042*
		Rotation	Moisture；Soil factors	0.022	0.341
		Soil factors	Moisture；Rotation	0.197	0.050
	Bulk soil	Moisture	Rotation；Soil factors	0.122	0.051
		Rotation	Moisture；Soil factors	0.280	0.013*
		Soil factors	Moisture；Rotation	0.220	0.036*
Fungi	Endosphere	Moisture	Rotation；Soil actors	−0.142	0.954
		Rotation	Moisture；Soil factors	0.358	0.007**
		Soil factors	Moisture；Rotation	0.429	0.001**
	Rhizosphere	Moisture	Rotation；Soil factors	0.063	0.150
		Rotation	Moisture；Soil factors	0.202	0.009**
		Soil factors	Moisture；Rotation	0.514	0.001**
	Bulk soil	Moisture	Rotation；Soil factors	-0.027	0.656
		Rotation	Moisture；Soil factors	0.489	0.002**
		Soil factors	Moisture；Rotation	0.360	0.001**

Note: * $P < 0.05$, ** $P < 0.01$, and ***$P < 0.001$.

2 Supplementary Figures

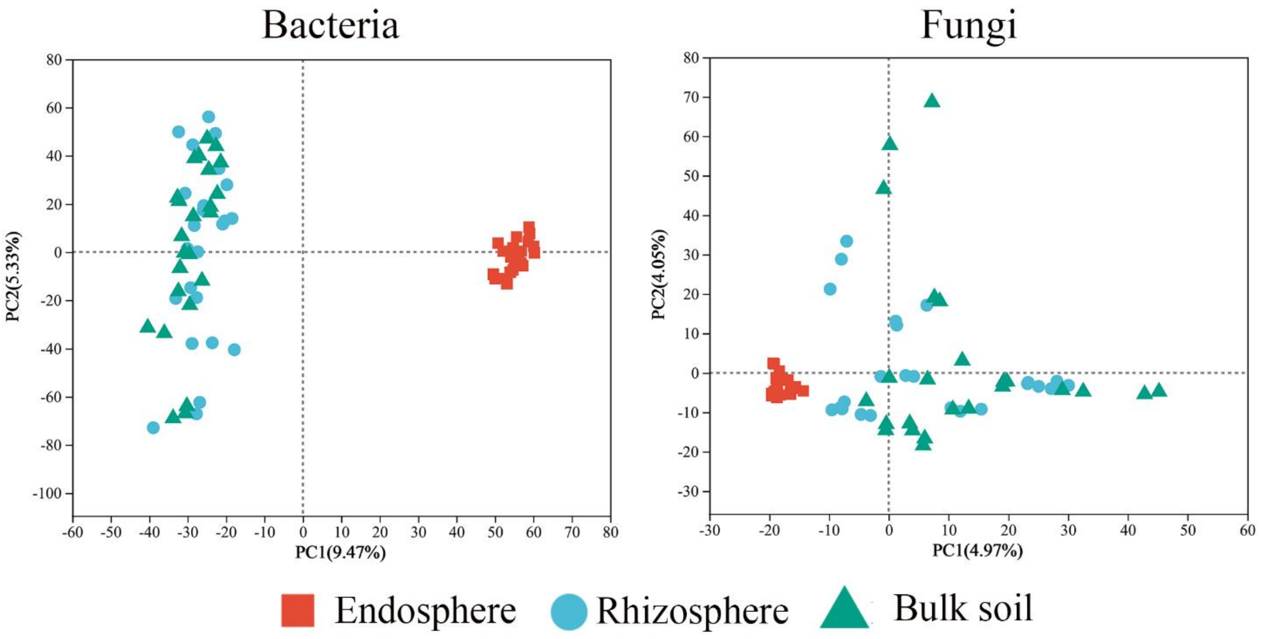

Supplementary Figure 1. Beta−diversity of the bacterial (A) and fungal (B) communities in endosphere, rhizosphere, and bulk soil of wheat visualized by Principal Component Analysis (PCoA) based on bray−Curtis distance metrics at the OTU level.

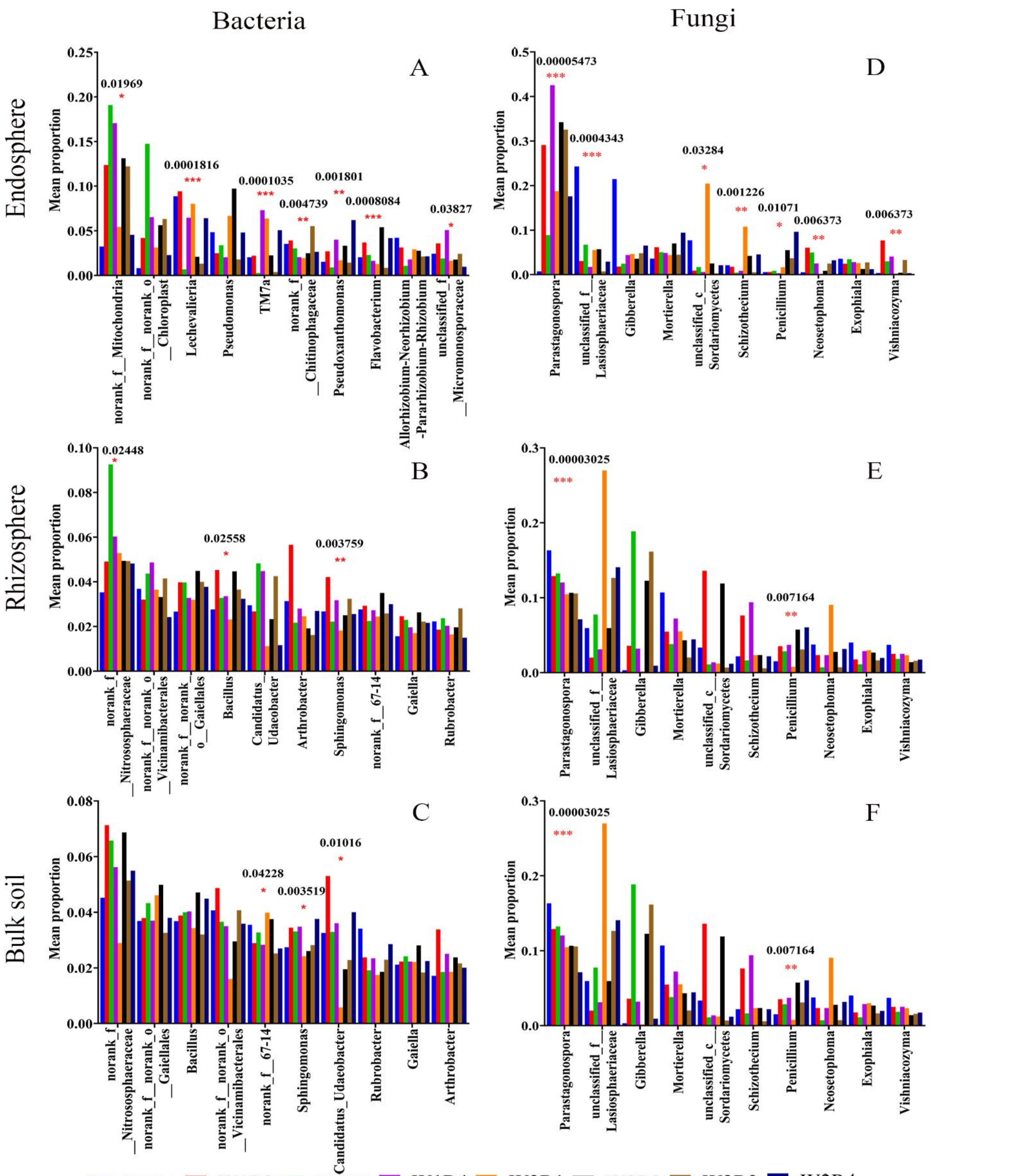

Supplementary Figure 2. Comparison of the dominant genus in the bacterial (A−C) and fungi (D−F) under the different drought stress−rotation patterns treatment. Statistical analysis was performed by the oneway ANOVA test. *$P < 0.05$, **$P < 0.01$, and ***$P \leq 0.001$.

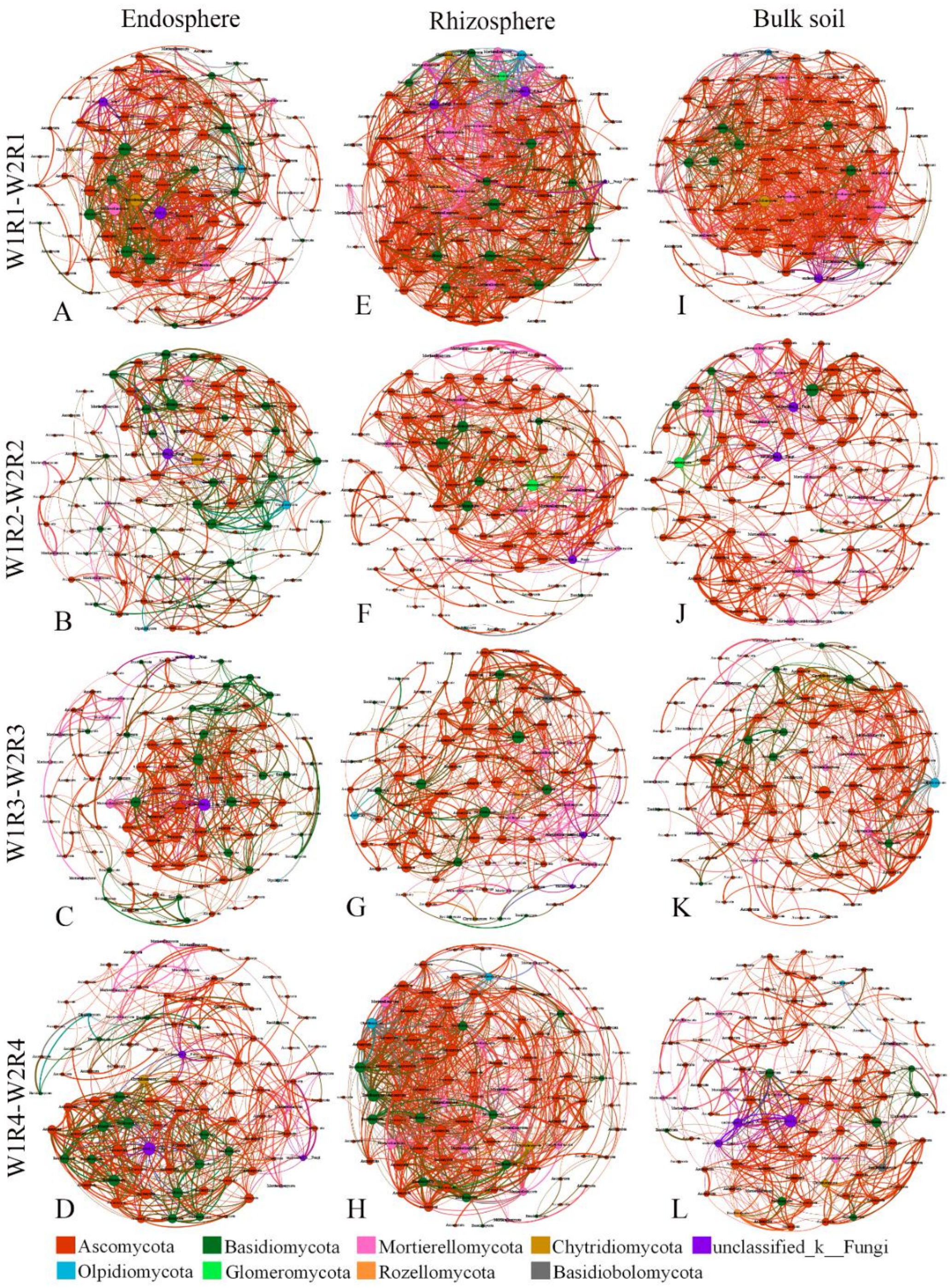

Supplementary Figure 3. Soil fungi co−occurrence networks of different drought stress−rotation model, including endosphere, rhizosphere and bulk soil. (A, E, I: W1R1−W2R1; B, F, J: W1R2−W2R2; C, G, K: W1R3−W2R3; D, H, L: W1R4−W2R4). Connections indicate significant correlation (Screening conditions: Spearman's $\rho > 0.8$, $P < 0.05$); The nodes are colored by phylum and represent an operational taxonomic unit (97% sequence identify threshold, OTU); the size of each node is proportional to the number of connections (degrees); the thickness of each connection between two nodes (edge) is proportional to the values of spearman's correlation coefficient.

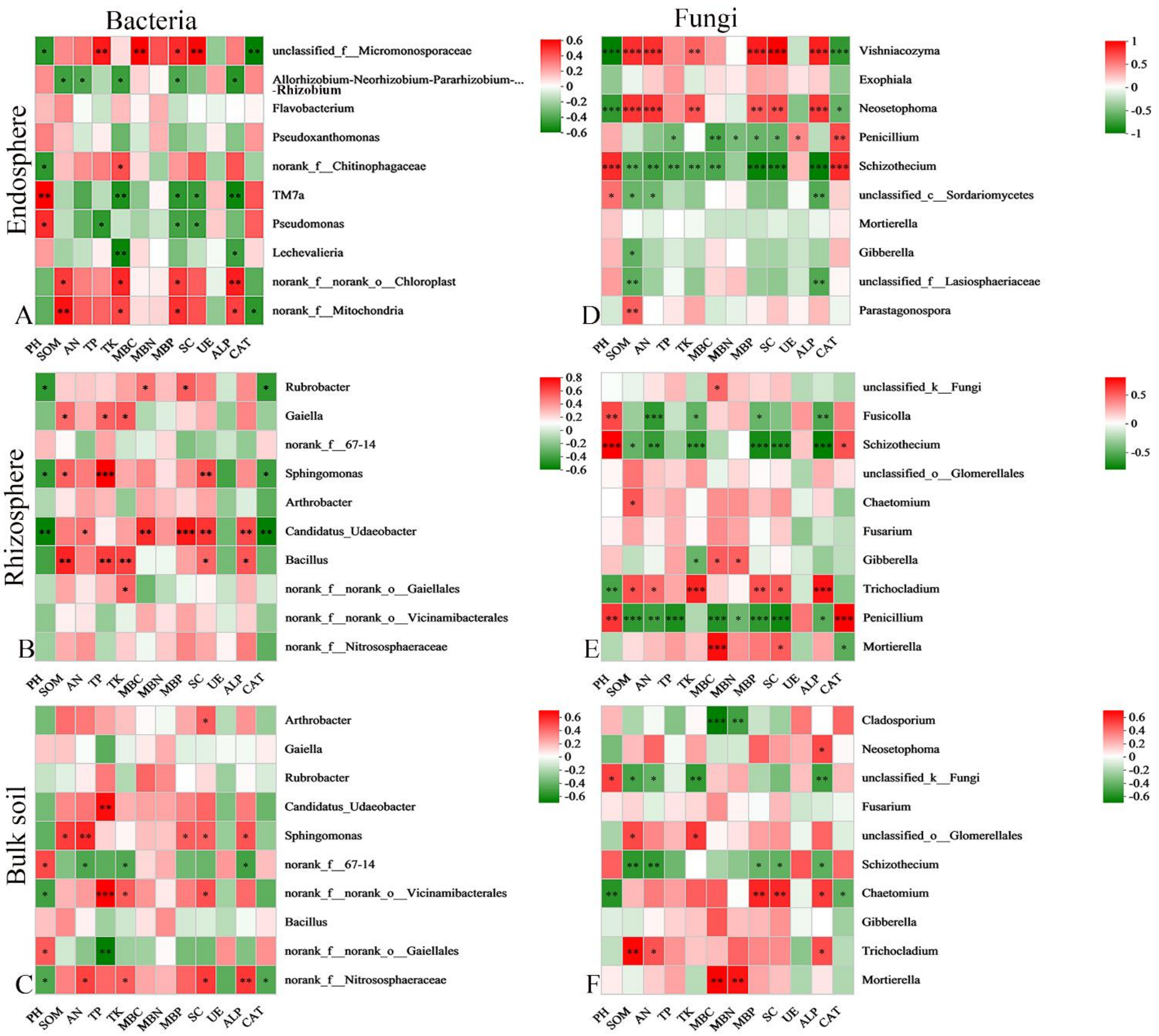

Supplementary Figure 4. Different drought stress−rotation patterns correlation heatmaps showing the wheat soil chemical variable in bacterial (A−C) and fungal (D−F) genus communities of a microbial classification relationship with the environmental variable, R−value, to show different colors in the picture. SOM, soil organic matter content; AN, soil available nitrogen content; TP, total phosphorus content; TK, total Soil total potassium content; MBC, Soil microbial biomass carbon; MBN, soil microbial biomass nitrogen; MBP, soil microbial biomass phosphorus; SC, soil sucrase; UE, Soil urease; ALP, soil alkaline phosphatase; CAT, soil catalase; * $P < 0.05$, ** $P < 0.01$, and ***$P < 0.001$.
